# A central limit theorem for the Euler method for SDEs with irregular drifts

**DOI:** 10.1007/s40072-026-00411-5

**Published:** 2026-04-06

**Authors:** Konstantinos Dareiotis, Máté Gerencsér, Khoa Lê

**Affiliations:** 1 University of Leeds, Leeds, UK; 2 TU Wien, Vienna, Austria

**Keywords:** Euler method, Stochastic sewing, Central limit theorem, 60H50, 60H35, 65C30

## Abstract

The goal of this article is to establish a central limit theorem for the Euler–Maruyama scheme approximating multidimensional SDEs with elliptic Brownian diffusion, under very mild regularity requirements on the drift coefficients. When the drift is Hölder continuous, we show that the limiting law of the rescaled fluctuations around the true solution is characterised as the unique solution of a hybrid Young–Itô differential equation. When the drift is bounded and has positive Sobolev regularity, this limit is characterised by the solution of a transformed SDE. Our results are extensions of the results of Jacod–Kurtz–Protter (1991, 1998) in which SDEs with differentiable coefficients were considered. To compensate for the lack of regularity of the drifts, we utilise the regularisation effect from the non-degenerate noise.

## Introduction

We consider the asymptotic error distributions for the Euler–Maruyama scheme for stochastic differential equations of the form

1.1 $$\begin{aligned} X_t=x_0+\int _0^tb(X_r)dr+\int _0^t \sigma (X_r)dB_r, \quad t\in [0,1]. \end{aligned}$$

Herein, $x_0\in {{\mathbb {R}}^d}$, d≥1, *B* is a standard Brownian motion in ${\mathbb {R}}^d$ on a filtered probability space $(\Omega ,\mathcal {F},\{\mathcal {F}_t\}_{t \in [0, 1]},\textbf{P})$, $b:{{\mathbb {R}}^d}\rightarrow {{\mathbb {R}}^d}$ and $\sigma :{{\mathbb {R}}^d}\rightarrow {\mathbb {R}}^{d\times d}$ are measurable functions. The Euler–Maruyama scheme for (1.1) is defined by

1.2 $$\begin{aligned} X^n_t=x_0+\int _0^tb(X^n_{\kappa _n(r)})dr+\int _0^t\sigma (X^n_{\kappa _n(r)})dB_r, \quad t\in [0,1], \end{aligned}$$

with the notation $\kappa _n(r)=\lfloor nr\rfloor /n$. When b,σ are continuously differentiable with linear growth, it is shown in [6, 8] that $X^n$ satisfies a central limit theorem. Namely, the probability law on $C([0, 1]; {\mathbb {R}}^d)$ of the process

1.3 $$\begin{aligned} V^n_t=\sqrt{n}(X_t-X^n_t) \end{aligned}$$

converges to the law of the solution $V=(V^{(1)},\ldots , V^{(d)})$ to the system of equations

1.4 $$\begin{aligned} \begin{aligned} V^{(\ell )}_t&=\int _0^t \partial _i b^{(\ell )} (X_r) V^{(i)}_r\,dr+\int _0^t \partial _j \sigma ^{(\ell , i)}(X_r)V^{(j)}_r\,dB^{(i)}_r\\&\quad +\frac{1}{\sqrt{2}}\int _0^t(\partial _j \sigma ^{(\ell , i)} \sigma ^{(j, k)})(X_r)\,dW^{(k, i)}_r, \quad \ell =1,\ldots ,d, \end{aligned} \end{aligned}$$

where we have adopted the Einstein summation convention over repeated indices. In (1.4), *W* is a new d×d-dimensional Brownian motion defined on an extension of $(\Omega ,\mathcal {F},\{\mathcal {F}_t\}_{t \in [0, 1]},\textbf{P})$ and is independent from the σ-field $\mathcal {F}$. For each (*k*, *i*), the process $W^{(k, i)}$ arises as the limit in law of the process

$$\begin{aligned} t\mapsto \sqrt{2n}\int _0^t (B^{(k)}_r-B^{(k)}_{\kappa _n(r)})dB^{(i)}_r. \end{aligned}$$

Our goal is to derive an analogous central limit theorem for equations whose drift is far from differentiable. Namely, we deal with the case when *b* possesses regularity of order $\alpha \in (0, 1)$, measured in either a Hölder or a Sobolev scale. In this setting, the limiting asymptotic distribution can not be directly characterised by (1.4) because it involves the derivative of *b*, which only exists in the distributional sense, and evaluating the distribution $\partial _i b^{(\ell )}$ at a given point is not a well-defined operation. We provide two possible ways of circumventing this problem.

When the drift possesses Hölder regularity of order $\alpha \in (0, 1)$, we make sense of the first integral in (1.4) as a Young integral ( [18]). This relies on the observation, which is a reoccurring one in the study of averaging along oscillatory processes, that even though $\partial _i b^{(\ell )}(X_r)$ is not well-defined, its integral with respect to *r* can be given a robust meaning. Indeed, by applying the stochastic sewing lemma from [12], one can define the process

1.5 $$\begin{aligned} L^{(\ell ,i)}_t=\int _0^t \partial _i b^{(\ell )}(X_r)\,dr \end{aligned}$$

and show that it is β-Hölder continuous, for any $\beta < (1+\alpha )/2$. Hence, if *V* is γ-Hölder continuous, for some $\gamma > 1 - (1+\alpha )/2=(1-\alpha )/2$, then integral of *V* against *L* can be defined as a Young integral. Then formally one has

1.6 $$\begin{aligned} \int _0^t \partial _i b^{(\ell )} (X_r) V^{(i)}_r\,dr=\int _0^tV^{(i)}\,dL^{(\ell ,i)}_r. \end{aligned}$$

Hence, the system (1.4) becomes a system of hybrid Young–Itô differential equations which will be shown to have a unique solution. Based upon this observation, the convergence of the laws of $V^n$ to the law of the solution of (1.4) can be extended in this case.

When *b* only possesses regularity in a Sobolev scale, the Hölder regularity of L deteriorates and we have not succeeded in treating (1.4) as a hybrid Young–Itô equation. Henceforth, we give an alternative approach. We rely on a Zvonkin type transformation that removes the distributional term. To be more precise, given $\ell \in \{1, \ldots , d\}$ and θ>0, let u be the solution to the partial differential equation (PDE)

1.7 $$\begin{aligned} \partial _t u^{(\ell )}+\frac{1}{2}a^{(i,j)}\partial ^2_{i,j} u^{(\ell )}+b\cdot \nabla u^{(\ell )}= \theta u^{(\ell )}-b^{(\ell )},\quad u^{(\ell )}(1,\cdot )=0. \end{aligned}$$

By applying Itô’s formula to $u(t, X_t)$, we obtain that

$$\begin{aligned} \int _0^t b(X_r) d r \!=\! u(0,X_0)-u(t, X_t) \!+\! \theta \!\int _0^t u(r, X_r) d r \!+\! \int _0^t (\partial _i u \sigma ^{(i,j)})(r, X_r) d B^{(j)}_r, \end{aligned}$$

and a similar expression for $\int _0^t b(X^n_r) d r$ (see (6.2)). Substituting these expressions into equations (1.1),(1.2), one obtains an equation for the process $V^n$. In the limit $n\rightarrow \infty$, this equation formally converges to the following equation, written for each coordinate ℓ=1,...,d,

1.8 $$\begin{aligned}&V^{(\ell )}_t- \partial _{j} u^{(\ell )}(t, X_t) V^{(j)}_t \end{aligned}$$

1.9 $$\begin{aligned}&\quad = \theta \int _0^t \, \partial _j u^{(\ell )}(r, X_r) V^{(j)}_r \, dr \nonumber \\&\qquad + \int _0^t \big ( \partial _j (\partial _\rho u^{(\ell )} \sigma ^{(\rho , i)}) (r, X_r) V^{(j)}_r + \partial _j \sigma ^{(\ell , i)}(X_r) V^{(j)}_r \big ) \, dB^{(i)}_r \nonumber \\&\qquad + \frac{1}{\sqrt{2}} \int _0^t\big ( ( I^{(\ell , \rho )}+ \partial _\rho u^{(\ell )} ) \partial _j \sigma ^{(\rho , i)}\sigma ^{(j, k)} \big )(r, X_r) \, dW_r^{(k, i)} . \end{aligned}$$

When θ is sufficiently large, the matrix I-∇u(t,x) is invertible so that (1.9) is, in fact, a linear Itô equation for the process $Z_t:=(I-\nabla u(t,X_t))V_t$ (see (6.14) herein). Thus, the system (1.4),(1.9) characterises (X,Z), and hence (X,V) uniquely. As in the previous case, this is sufficient to obtain the convergence in probability laws of $V^n$ to the law of V, which, in this case, is characterised by equation (1.9).

Presumably, the latter approach could be applied equally well to treat the case of Hölder continuous drifts. Nevertheless, it relies crucially on the validity of Itô formulas and therefore is limited to SDEs driven by Brownian motion. The former approach takes advantage of recent progresses in regularisation by noise problem. It is based upon the stochastic sewing lemma from [12] and the analysis of the hybrid Young–Itô differential equation of the type (1.4). Because Itô calculus is not essesntial in this approach, it could be beneficial in other settings, such as SDEs driven by fractional Brownian motion.

The paper is organised as follows: we formulate our main results precisely under Hölder and Sobolev regularity conditions respectively in Sections 2 and 3. In Section 4, we obtain some quadrature-type estimates which show that certain terms vanish in the limit $n\rightarrow \infty$. The proofs of the main results are presented in Sections 5 and 6. The Appendix contains some auxiliary technical results.

### Notation

Let us introduce some notation that will be frequently used throughout the paper. For any function f:Q→V, where $Q\subset {\mathbb {R}}^k$ is a Borel set and (V,|·|) is a normed space, with the notation $\mathbb {N}_0:= \mathbb {N}\cup \{0\}$, let us introduce the (semi-)norms

$$\begin{aligned}&\Vert f\Vert _{C^0(Q,V)}=\sup _{x\in Q}|f(x)|\,; \\&\,[f]_{C^\gamma (Q,V)}=\sum _{\begin{array}{c} \ell \in \mathbb {N}_0^k\\ |\ell |_1={\hat{\gamma }} \end{array}}\sup _{x\ne y\in Q}\frac{|\partial ^{\ell }f(x)-\partial ^{\ell }f(y)|}{|x-y|^{{\bar{\gamma }}}},\\&\gamma>0,\,\gamma ={\hat{\gamma }}+{\bar{\gamma }},\,{\hat{\gamma }}\in \mathbb {N}_0,{\bar{\gamma }}\in (0,1];\\&\Vert f\Vert _{C^\gamma (Q,V)}=\sum _{\begin{array}{c} \ell \in \mathbb {N}_0^k\\ |\ell |_1< \gamma \end{array}} \Vert \partial ^\ell f\Vert _{C^0(Q,V)}+[f]_{C^\gamma (Q,V)}, \gamma >0. \end{aligned}$$

In the above $|\ell |_1=\ell _1+\ldots +\ell _k$ if $\ell =(\ell _1,\ldots ,\ell _k)\in \mathbb {N}_0^k$. By $C^\gamma (Q,V)$ we denote the space of all measurable functions f:Q→V such that $\Vert f\Vert _{C^\gamma (Q,V)}<\infty$. Frequently the target space will be dropped from the notation, if this does not cause confusion. For example, we will write $C^\gamma ({\mathbb {R}}^d)$ in place of $C^\gamma ({\mathbb {R}}^d; V)$ if the target space *V* is understood from the context. Notice that $C^0$ denotes the collection of bounded functions. Similarly, for $k \in \mathbb {N}$, $C^k$ functions are bounded (k-1)-times continuously differentiable and the derivatives of (k-1)-order are Lipschitz continuous. For $\gamma \in {\mathbb {R}}_+ \setminus \mathbb {N}_0$, $C^\gamma$ are of course the usual Hölder spaces. For $\gamma \in (0, 1)$, we will denote by $C^{\gamma +}$ the closure of $C^\infty := \cap _{k=1}^\infty C^k$ in $C^\gamma$. It is easy to see that $C^{\gamma +\varepsilon }\hookrightarrow C^{\gamma +}\hookrightarrow C^{\gamma }$ for every $\gamma \in (0,1)$ and ε>0. For γ<0, we denote by $C^\gamma ({\mathbb {R}}^d)$ the space of all tempered distributions *f* such that

$$\begin{aligned} \Vert f\Vert _{C^\gamma ({\mathbb {R}}^d)} := \sup _{t \in (0,1)} t^{-\gamma /2} \Vert \mathcal {P}_t f\Vert _{C^0({\mathbb {R}}^d)} < \infty , \end{aligned}$$

where $(\mathcal {P}_t) _{t \ge 0}$ is the heat semigroup associated to the standard Gaussian kernel $p_t(x)=(2 \pi t)^{-d/2}e^{-|x|^2/(2t)}$. For $p\in [1,\infty )$ and a probability space $(\Omega , \mathcal {F}, \textbf{P})$, we write $L_p(\Omega ; V)$ for the space of V-valued random variables η such that $\Vert \eta \Vert _{L_p(\Omega ;V)}:=(\textbf{E}|\eta |^p)^{1/ p}<\infty$. As previously, we write $L_p(\Omega )$ in place of $L_p(\Omega ;V)$ if the target space V is understood from the context. When a filtration $(\mathcal {F}_t)$ is specified, we write $\textbf{E}^s$ for the conditional expectation with respect to $\mathcal {F}_t$, i.e. $\textbf{E}(\cdot |\mathcal {F}_t)$. For a two parameter function $A_{s,t}$, we put $\delta A_{s,u,t}=A_{s,t}-A_{s,u}-A_{u,t}$. For a vector/ matrix *y*, its transpose is denoted by $y^*$.

Finally, a notational convention: in proofs of statements we use the shorthand f≲g to mean that there exists a constant *N* such that f≤Ng, and that *N* does not depend on any other parameters than the ones specified in the statement.

## Formulation and main results for Hölder drift

We begin with the presentation of our results in the case that *b* possesses Hölder regularity. The proofs are postponed to Section 5.

### Assumption 2.1

The coefficient σ belongs to $C^2({\mathbb {R}}^d; {\mathbb {R}}^{d \times d})$ and there exists λ>0 such that $|y^* (\sigma \sigma ^*)(x) y|\ge \lambda ^2|y|^2$ for all $x,y\in {\mathbb {R}}^d$.

### Assumption 2.2

For some $$\alpha \in (0, 1)$$, $$b \in C^{\alpha +}({\mathbb {R}}^d; {\mathbb {R}}^d)$$.

We will mostly be dealing with solutions in a weak sense. To be more precise, by a solution of (1.1) we mean the following:

### Definition 2.3

A solution of (1.1) is a collection $\{ (\Omega , \mathcal {F}, \mathbb {F}, \textbf{P}), X, B\}$ such that

(i) $(\Omega , \mathcal {F}, \textbf{P})$ is a probability space with a filtration $\mathbb {F}=\{ \mathcal {F}_t\}_{t \in [0, 1]}$.
(ii) *X* is an ${\mathbb {R}}^d$-valued process on Ω, adapted to $\mathbb {F}$.
(iii) *B* is an ${\mathbb {R}}^d$-valued $\mathbb {F}$-Wiener process on Ω.
(iv) Equality (1.1) is satisfied $\textbf{P}$-almost surely, for all t∈[0,1].

It is well-known that under the above assumptions, equation (1.1) admits a unique solution (see, [17]). Next, we want to give a meaning to equation (1.4) and its solution. To do this, we need the following proposition which provides rigorous meaning to the process L in (1.5).

### Proposition 2.4

Let Assumptions 2.1 and 2.2 hold and let $\{ (\Omega , \mathcal {F}, \mathbb {F}, \textbf{P}), X, B\}$ be a solution of (1.1). Then, there exists a bounded linear operator

$$\begin{aligned} C^{\alpha +}({\mathbb {R}}^d) \ni f \mapsto (Q^Xf)_{t \in [0, 1]} \in C^{(1+\alpha )/2}([0, 1]; L_2(\Omega ; {\mathbb {R}}^d )) \end{aligned}$$

such that

(i) For all $f \in C^{\alpha +}({\mathbb {R}}^d)$, the process $(Q^Xf)_{t \in [0, 1]}$ is $\mathbb {F}$-adapted.
(ii) For all $f \in C^1({\mathbb {R}}^d)$, with probability one,
  $$\begin{aligned} (Q^Xf)_t = \int _0^t \nabla f(X_s) \, ds, \quad \text {for all } t \in [0, 1]. \end{aligned}$$
(iii) For all p≥1, there exists a constant N=N(p,α,d) such that
  $$\begin{aligned} \Vert Q^Xf\Vert _{C^{(1+\alpha )/2}([0, 1]; L_p(\Omega ; {\mathbb {R}}^d))} \le N \Vert f\Vert _{C^{\alpha }({\mathbb {R}}^d)}. \end{aligned}$$

### Remark 2.5

Let $f \in C^{\alpha +}({\mathbb {R}}^d)$. By (iii) of the above result and Kolmogorov’s continuity theorem (see Lemma A.2 below), it follows that for all p≥1 and $\alpha ' \in (0, (1+\alpha )/2)$, there exists $N=N(p,\alpha ,\alpha ', d)$ such that

$$\begin{aligned} \Vert Q^Xf \Vert _{L_p(\Omega ; C^{\alpha '}([0,1]; {\mathbb {R}}^d))} \le N \Vert f\Vert _{C^\alpha ({\mathbb {R}}^d)}. \end{aligned}$$

In particular, if $Z \in C^\beta ([0, 1]; {\mathbb {R}}^d)$, then integrals of the form $\int Z_r d (Q^Xf)_r$ can be defined almost surely as Young integrals provided that $\beta >(1-\alpha )/2$.

### Remark 2.6

If $f \in C^{\alpha +}({\mathbb {R}}^d; {\mathbb {R}}^d)$ we will denote by $Q^Xf$ the ${\mathbb {R}}^{d\times d}$-valued process given by $(Q^Xf)^{(i, j)}:=(Q^Xf^{(i)})^{(j)}$, $i, j \in \{1, ..., d\}$.

### Definition 2.7

A solution of the system (1.1),(1.4) is a collection $\{ (\Omega , \mathcal {F}, \mathbb {F}, \textbf{P}), X, V, B, W\}$ such that

(i) $\{(\Omega , \mathcal {F}, \mathbb {F}, \textbf{P}), X, B\}$ is a solution of (1.1).
(ii) *W* is an ${\mathbb {R}}^{d\times d}$-valued $\mathbb {F}$-Wiener process on Ω, independent of (*X*, *B*).
(iii) *V* is an $\mathbb {F}$-adapted process and there exists $\beta > (1-\alpha )/2$ such that $\textbf{P}\big ( V \in C^\beta ([0, 1]; {\mathbb {R}}^d)\big ) =1$.
(iv) For all $\ell \in \{1, ..., d\}$,
  2.1 $$\begin{aligned} V^{(\ell )}_t&= \int _0^t V^{(j)}_r\,d(Q^X b )^{( \ell ,j)}_ r+ \int _0^t \partial _j \sigma ^{(\ell , i)}(X_r)V^{(j)}_r\,dB^{(i)}_r \nonumber \\&\quad + \frac{1}{\sqrt{2}}\int _0^t(\partial _j \sigma ^{(\ell , i)}\sigma ^{(j, k)} )(X_r)\,dW^{(k, i)}_r, \end{aligned}$$
  $\textbf{P}$-almost surely, for all t∈[0,1].

To ease the notation, we will use the shorthand version of (2.1)

$$\begin{aligned} V_t= \int _0^t \,d(Q^Xb )_r V_r +\int _0^t \nabla \sigma (X_r)V_r\,dB_r +\frac{1}{\sqrt{2}}\int _0^t \nabla \sigma \sigma (X_r)\,dW_r. \end{aligned}$$

For the following theorems, let us introduce the notation

$$\begin{aligned} \mathcal {C}:= \big (C([0,1]; {\mathbb {R}}^d)\big )^3 \times C([0,1]; {\mathbb {R}}^{d\times d}). \end{aligned}$$

### Theorem 2.8

There exists a solution $$\{ (\Omega , \mathcal {F}, \mathbb {F}, \textbf{P}), X, V, B, W\}$$ of the system (1.1),(1.4). Moreover, if $$\{ (\Omega ', \mathcal {F}', \mathbb {F}', \textbf{P}'), X', V', B', W'\}$$ is another solution, then the laws of (*X*, *V*, *B*, *W*) and $\left(X^{\prime },V^{\prime },B^{\prime },W^{\prime }\right)$ on $$\mathcal {C}$$ coincide.

The law of the unique solution (*X*, *V*, *B*, *W*) of the system (1.1),(1.4) will be denoted by μ. We are now in position to state our main result for Hölder drifts.

### Theorem 2.9

Let Assumptions 2.1 and 2.2 and let $\{ (\Omega , \mathcal {F}, \mathbb {F}, \textbf{P}), X, B\}$ be a solution of (1.1). Define the processes $X^n, W^n$, and $V^n$ by

$$\begin{aligned} dX^n_t&= b(X^n_{\kappa _n(t)})dt+ \sigma (X^n_{\kappa _n(t)})dB_t, \qquad X^n_0=x_0,\\ dW^n_t&= \sqrt{2n} (B_t-B_{\kappa _n(t)}) \otimes \, dB_t, \qquad W^n_0=0,\\ V^n_t&= \sqrt{n}(X_t-X^n_t). \end{aligned}$$

Then, the law of $(X, V^n, B, W^n)$ on $\mathcal {C}$ converges weakly to μ.

### Remark 2.10

The endpoint case α=0, corresponding to merely bounded and measurable *b*, appears to be a quite challenging problem, in fact it is not even clear whether the factor $\sqrt{n}$ is the correct one. Even a much simpler problem, determining the sharp rate of convergence of

$$\begin{aligned} I_n:=\int _0^1 \big (b(B_t)-b(B_{\kappa _n(t)})\big ) \,dt \end{aligned}$$

for general bounded measurable function *b* is an open question. The best known rate is the bound $\Vert I_n\Vert _{L^p(\Omega )}\lesssim n^{-1/2}\sqrt{\log n}$ for all p≥2. The case p=2 is established in [2] by direct estimations while the case p>2 is established in [13] using the known estimate for p=2 and the John–Nirenberg inequality for stochastic processes of bounded mean oscillation.

## Formulation and main results for Sobolev drift

In this section, we present our results in the case that *b* possesses positive regularity in a Sobolev scale. More precisely, Assumption 2.2 is replaced by the following Assumption 3.1, while Assumption 2.1 remains in force. The proofs are postponed to Section 6.

### Assumption 3.1

For some α>0 and m≥1 the coefficient *b* belongs to $W^\alpha _m({\mathbb {R}}^d; {\mathbb {R}}^d)$, that is,

$$\begin{aligned} \Vert b\Vert _{W^\alpha _m({\mathbb {R}}^d; {\mathbb {R}}^d)}^m:=\int _{{\mathbb {R}}^d} |b(x)|^m\, dx + \int _{{\mathbb {R}}^d}\int _{{\mathbb {R}}^d}\frac{|b(x)-b(y)|^m}{|x-y|^{d+\alpha m}}\,dx\,dy<\infty . \end{aligned}$$

Moreover, *b* is bounded.

### Remark 3.2

Notice that if Assumption 3.1 is satisfied, then for any $m^{\prime }\geq 1$ there exists $$\alpha ' \in (0, \alpha )$$ such that $$b \in W^{\alpha '}_{m'}({\mathbb {R}}^d; {\mathbb {R}}^d)$$. Therefore, without loss of generality we can assume that $$m \ge \max \{2, d\}$$.

### Remark 3.3

The boundedness condition on b is neccessary for our method. For unbounded drift, the Euler–Maruyama scheme is not well-behaved and one usually resorts to a tamed Euler–Maruyama scheme, see [5, 14].

The limiting distribution in this case will be characterised by means of the Zvonkin transform. In order to proceed with the precise statement, we will need some standard facts from PDE theory. Under Assumption 3.1, *b* belongs to $L_{p_0}({{\mathbb {R}}^d}; {\mathbb {R}}^d)$ for all $p_0\ge m$. Given $\ell \in \{1, \ldots , d\}$ and θ>0, we consider the PDE (1.7).

Under Assumptions 2.1 and 3.1, by [10, Theorem 10, p.123] there exists a unique solution $u^{(\ell )}\in W^{1,2}_{p_0}((0, 1) \times {\mathbb {R}}^d)$, where the latter denotes the space of all measurable, locally integrable functions $v :[0, 1] \times {\mathbb {R}}^d \rightarrow {\mathbb {R}}$ such that

$$\begin{aligned} \Vert v\Vert ^{p_0} _{W^{1,2}_{p_0}((0, 1) \times {\mathbb {R}}^d)}:= \int _0^1 \int _{{\mathbb {R}}^d} | v|^{p_0}+|\nabla v|^{p_0}+| \nabla ^2 v |^{p_0}+ | \partial _tv|^{p_0} \, dx dt < \infty . \end{aligned}$$

Additionally, by [10, Theorem 1, p.120] there exist positive constants *N*, $\theta _0$ depending only in $d, p_0, \lambda , \Vert \sigma \Vert _{C^2}$ and $\Vert b\Vert _{C^0}$ such that, the estimate

3.1 $$\begin{aligned} \Vert u^{(\ell )}\Vert _{W^{1,2}_{p_0}((0, 1) \times {\mathbb {R}}^d)} \le N \Vert b^{(\ell )} \Vert _{L_{p_0}((0, 1) \times {\mathbb {R}}^d)} \end{aligned}$$

holds. Notice that the solution does not depend on the choice of $p_0$ and the above estimates are valid for all $p_0 \ge m$ for the same solution. Moreover, for any θ sufficiently large, we have

3.2 $$\begin{aligned} \Vert \nabla u^{(\ell )}\Vert _{C^0([0, 1] \times {\mathbb {R}}^d)} \le N \theta ^{-1/2}, \end{aligned}$$

with $N=N(\lambda , d, \Vert \sigma \Vert _{C^2}, \Vert b\Vert _{C^0}, \Vert b\Vert _{L_m})$ (see, for example [3, Lemma 2.10]). It is also known (see, [20, Lemma 2.1]) that (3.1) implies that for each $\gamma \in (0, 1)$, if $p_0$ is large enough, then

3.3 $$\begin{aligned} \Vert \nabla u ^{(\ell )} \Vert _{C^{\gamma /2, \gamma }([0, 1]\times {\mathbb {R}}^d)} \le N \Vert b^{(\ell )} \Vert _{L_{p_0}({\mathbb {R}}^d)}, \end{aligned}$$

with $N= N(d, p_0, \lambda , \Vert \sigma \Vert _{C^2}, \Vert b\Vert _{C^0})$. Finally, since in addition $b \in W^\alpha _m({\mathbb {R}}^d ; {\mathbb {R}}^d )$, it also follows (see Lemma A.3) that

3.4 $$\begin{aligned} \Vert u^{(\ell )}\Vert _{L_{p_0}((0, 1); W^{2+\alpha }_m ({\mathbb {R}}^d))} \le N \Vert b^{(\ell )}\Vert _{W^\alpha _m({\mathbb {R}}^d)}, \end{aligned}$$

with $$N=N(\alpha , \lambda , d, \Vert \sigma \Vert _{C^2}, \Vert b\Vert _{C^0}, \Vert b\Vert _{L_m}, \theta )$$, where

$$\begin{aligned} \Vert u^{(\ell )}\Vert _{L_{p_0}((0, 1); W^{2+\alpha }_m ({\mathbb {R}}^d))}:= \Vert u^{(\ell )}\Vert _{L_{p_0}((0, 1); W^{2}_m ({\mathbb {R}}^d))}+ \Vert \nabla ^2 u^{(\ell )}\Vert _{L_{p_0}((0, 1); W^\alpha _m ({\mathbb {R}}^d))}. \end{aligned}$$

Let us set $u=(u^{(1)}, ..., u^{(d)})$ and fix a θ sufficiently large, so that the matrix I-∇u(t,x) is invertible and both I-∇u(t,x) and $(I- \nabla u (t, x))^{-1}$ are bounded uniformly in (*t*, *x*), which can be done because of (3.2). With this setting, we consider (1.1) coupled with (1.9). As previously, to ease the notation, we can also use the shorthand version of (1.9)

3.5 $$\begin{aligned} (I-\nabla u(t,X_t))V_t&= \theta \int _0^t\nabla u(r,X_r)V_rdr +\int _0^t[\nabla \big ( (\nabla u + I ) \sigma \big ) ](r,X_r)V_rdB_r \nonumber \\&\quad +\frac{1}{\sqrt{2}}\int _0^t(I+\nabla u)\nabla \sigma \sigma (r,X_r) dW_r, \end{aligned}$$

where the precise meaning of multiplication of tensors/matrices is understood by (1.9).

### Definition 3.4

A solution of the system (1.1),(1.9) is a collection $\{ (\Omega , \mathcal {F}, \mathbb {F}, \textbf{P}), X, V, B, W\}$ such that

(i) $\{(\Omega , \mathcal {F}, \mathbb {F}, \textbf{P}), X, B\}$ is a solution of (1.1).
(ii) *W* is an ${\mathbb {R}}^{d\times d}$-valued $\mathbb {F}$-Wiener process on Ω, independent of (*X*, *B*).
(iii) *V* is a continuous $\mathbb {F}$-adapted process.
(iv) Equation (1.9) is satisfied $\textbf{P}$-almost surely, for all t∈[0,1].

### Theorem 3.5

There exists a solution $$\{ (\Omega , \mathcal {F}, \mathbb {F}, \textbf{P}), X, V, B, W\}$$ of the system (1.1),(1.9) . Moreover, if $$\{ (\Omega ', \mathcal {F}', \mathbb {F}', \textbf{P}'), X', V', B', W'\}$$ is another solution, then the laws of (*X*, *V*, *B*, *W*) and $\left(X^{\prime },V^{\prime },B^{\prime },W^{\prime }\right)$ on $$\mathcal {C}$$ coincide.

The law of the unique solution (*X*, *V*, *B*, *W*) of the system (1.1),(1.9) will be denoted by $\tilde{\mu }$.

### Theorem 3.6

Let Assumptions 2.1 and 3.1 hold. With the notation of Theorem 2.9 we have that the laws $(X, V^n, B, W^n)$ converge weakly to $\tilde{\mu }$.

## Quadrature estimates

In this section, we prove some quadrature-type estimates which will be used for the proofs of our main results. More precisely, for functions g=g(t,x) and f=f(x), and $(s,t)\in [0,1]_{\le }^2:= \{ (s, t) \in [0, 1]^2; s \le t \}$, we estimate moments of the following integral

$$\begin{aligned} \int _s^t g (r, X^n_r)&\big ( f(X^n_r)-f(X^n_{\kappa _n(r)})\big )\, dr. \end{aligned}$$

In the results of the current section, when one of *g*, *f* has positive Sobolev regularity and the other one has positive Hölder regularity, we show that moments of all positive orders of the above integral is of order $n^{-(1+\varepsilon )/2}|t-s|^{1/2}$ for some ε>0. Integrals of this type appear in the equations for $V^n$ (see (5.18) and (6.18) below) and our quadrature-type estimates show that they vanish in the limiting equation for V.

We first recall the statement of the stochastic sewing lemma from [12].

### Lemma 4.1

Fix p≥2 and let *A* be a function from $[0,1]_\le :=\{(s,t)\in [0,1]^2:s\le t\}$ to $L_p(\Omega )$ such that $A_{s,t}$ is $\mathcal {F}_t$-measurable for all $(s,t)\in [0,1]_{\le }$. Suppose that there exist constants $\varepsilon _1,\varepsilon _2>0$ and $C_1,C_2<\infty$ such that the bounds

4.1 $$\begin{aligned} \Vert A_{s,t}\Vert _{L_p(\Omega )}&\le C_1 |t-s|^{1/2+\varepsilon _1}, \quad \Vert \textbf{E}^s\delta A_{s,u,t}\Vert _{L_p(\Omega )}&\le C_2 |t-s|^{1+\varepsilon _2} \end{aligned}$$

hold for every $(s,t)\in [0,1]_\le$. Then there exists a unique function $\mathcal {A}:[0,1]\rightarrow L_p(\Omega )$ such that $\mathcal {A}_0=0$, $\mathcal {A}_t$ is $\mathcal {F}_t$-measurable for each t∈[0,1], and the following bounds hold for some constants $K_1,K_2$ and for all $(s,t) \in [0, 1] _{\le }$

4.2 $$\begin{aligned} \Vert \mathcal {A}_t-\mathcal {A}_s-A_{s,t}\Vert _{L_p(\Omega )}&\le K_1|t-s|^{1/2+\varepsilon _1}+K_2 |t-s|^{1+\varepsilon _2}, \end{aligned}$$

4.3 $$\begin{aligned} \Vert \textbf{E}^s(\mathcal {A}_t-\mathcal {A}_s-A_{s,t})\Vert _{L_p(\Omega )}&\le K_2|t-s|^{1+\varepsilon _2}. \end{aligned}$$

Moreover, there exists a constant *N* depending only on $p, \varepsilon _1, \varepsilon _2$ such that for all $(s,t) \in [0, 1] _{\le }$ we have

4.4 $$\begin{aligned} \Vert \mathcal {A}_t-\mathcal {A}_s\Vert _{L_p(\Omega )}\le NC_1 |t-s|^{1/2+\varepsilon _1}. \end{aligned}$$

### Lemma 4.2

Let $b \in C^0({\mathbb {R}}^d; {\mathbb {R}}^d)$, σ satisfy Assumption 2.1, and let p≥2, $\theta \in (0, 1)$, $\rho \in [1, \infty ]$. Suppose that *X*, $X^n$ satisfy (1.1) and (1.2), respectively. Then, there exist $q_0 \ge 1$, $\beta _0 \in (0, 1)$, and ε>0 such that for all $q \ge q_0, \beta \ge \beta _0$, $g \in L_q( (0, 1) ; C^\beta ({\mathbb {R}}^d))$, $f \in C^0({\mathbb {R}}^d) \cap W^\theta _\rho ({\mathbb {R}}^d)$, $(s,t ) \in [0,1]_{\le }^2$, and $n\in \mathbb {N}$, one has the bound

4.5 $$\begin{aligned} \big \Vert \int _s^t g (r, X^n_r)&\big ( f(X^n_r)-f(X^n_{\kappa _n(r)})\big )\, dr\big \Vert _{L_p(\Omega )} \nonumber \\&\le N \Vert g\Vert _{L^q((0, 1);C^\beta ({\mathbb {R}}^d))} \big (\Vert f\Vert _{W^{\theta }_\rho ({\mathbb {R}}^d)}+\Vert f\Vert _{C^0({\mathbb {R}}^d)}\big ) n^{-(1+\varepsilon )/2}|t-s|^{1/2}, \end{aligned}$$

where *N* is a constant depending only on $\theta , \rho , p, \Vert b\Vert _{C^0}, \Vert \sigma \Vert _{C^2}, d$, and λ.

### Proof

Let us denote by $\bar{X}^n$ the Euler scheme corresponding to b≡0. By Girsanov’s transform, it suffices (see [3, Corollary 3.2]) to prove the claim for $\bar{X}^n$ . Estimate (4.5) is obtained by interpolation between the cases $f \in C^0({\mathbb {R}}^d) \cap L_\rho ({\mathbb {R}}^d)$ and $f \in C^0({\mathbb {R}}^d) \cap W^k_\rho ({\mathbb {R}}^d)$ for some large integer *k*. The estimate corresponding to each class is obtained through the stochastic sewing lemma.

*Step 1.* Suppose that $f \in C^0({\mathbb {R}}^d) \cap L_\rho ({\mathbb {R}}^d)$. We set

$$\begin{aligned} A_{s, t} = \textbf{E}^s \int _s^tg(r,\bar{X}^n_s) \big ( f(\bar{X}^n_r)-f({\bar{X}}^n_{\kappa _n(r)})\big )\, dr,\\ \mathcal {A}_t = \int _0^t g(r,\bar{X}^n_r) \big ( f(\bar{X}^n_r)-f({\bar{X}}^n_{\kappa _n(r)})\big )\, dr. \end{aligned}$$

It has been shown in [3] (see the proof of Lemma 3.1 therein) that for all $\gamma \in (0,1/ 2 )$ and r>s we have

4.6 $$\begin{aligned} |\textbf{E}^s\big ( f(\bar{X}^n_r)-f(\bar{X}^n_{\kappa _n(r)})\big )| \lesssim n^{-1/2+\gamma }\Vert f\Vert _{C^0({\mathbb {R}}^d)} |r-s|^{-1/2+\gamma }. \end{aligned}$$

Using the regularity of *g* we see that

$$\begin{aligned} \Vert A_{s, t}\Vert _{L_p(\Omega ) }&\lesssim n^{-1/2+\gamma }\Vert f\Vert _{C^0({\mathbb {R}}^d)} \int _s^t \Vert g(r, \cdot )\Vert _{C^\beta ({\mathbb {R}}^d)} |r-s|^{-1/2+\gamma } \, dr\\&\lesssim n^{-1/2+\gamma } \Vert g\Vert _{L^q((0, 1);C^\beta ({\mathbb {R}}^d))} \Vert f\Vert _{C^0({\mathbb {R}}^d)} |t-s|^{(1+\gamma )/2}, \end{aligned}$$

provided that *q* is sufficiently large. Moreover, we have

$$\begin{aligned} \textbf{E}^s \delta A_{s, u, t} = \textbf{E}^s \int _u^t ( g(r,\bar{X}^n_s)-g(r,\bar{X}^n_u) ) \textbf{E}^u \big ( f(\bar{X}^n_r)-f({\bar{X}}^n_{\kappa _n(r)})\big )\, dr. \end{aligned}$$

Using again (4.6) combined with the fact that $g \in L_q( (0, 1); C^\beta ({\mathbb {R}}^d))$ and that ${\bar{X}}^n_s -{\bar{X}}^n_u$ is of order $| s-u|^{1/2}$ in $L_p(\Omega )$ uniformly in *n*, it gives

4.7 $$\begin{aligned}&\Vert \textbf{E}^s \delta A_{s, u, t}\Vert _{L_p(\Omega )} \nonumber \\&\quad \lesssim \Vert f\Vert _{C^0} n^{-1/2+\gamma } |u-s|^{\beta /2} \int _u^t \Vert g(r, \cdot )\Vert _{C^\beta } |r-u|^{-1/2+\gamma } \, dr \nonumber \\&\quad \lesssim \Vert f\Vert _{C^0({\mathbb {R}}^d)} \Vert g\Vert _{L^q((0, 1);C^\beta ({\mathbb {R}}^d))} n^{-1/2+\gamma } |u-s|^{\beta /2} |t-u|^{-1/2+\gamma +1/q^*} \nonumber \\&\quad \lesssim \Vert f\Vert _{C^0({\mathbb {R}}^d)} \Vert g\Vert _{L^q((0, 1);C^\beta ({\mathbb {R}}^d))} n^{-1/2+\gamma } |t-s|^{-1/2+\gamma +1/q^*+\beta /2}, \end{aligned}$$

where $q^*=q/(q-1)$. Now we fix $\beta _0 \in (0, 1)$ sufficiently close to 1 and $q_0$ sufficiently large so that

4.8 $$\begin{aligned} -1/2+\gamma +1/q_0^*+{\beta _0}/2 >1, \end{aligned}$$

and notice that the same relation holds for all $\beta \ge \beta _0$ and $q \ge q_0$. Moreover, by using the boundedness of *f* and the regularity of *g*, it is easy to see that

$$\begin{aligned} \Vert \mathcal {A}_t&-\mathcal {A}_s- A_{s, t}\Vert _{L_p(\Omega )} \\&= \big \Vert \int _s^tg(r,\bar{X}^n_r) \big ( f(\bar{X}^n_r)-f({\bar{X}}^n_{\kappa _n(r)})\big )\, dr\\&\quad - \textbf{E}^s \int _s^tg(r,\bar{X}^n_s) \big ( f(\bar{X}^n_r)-f({\bar{X}}^n_{\kappa _n(r)})\big )\, dr \big \Vert _{L_p(\Omega )} \\&\le 4 \Vert f\Vert _{C^0} \int _s^t \Vert g(r, \cdot )\Vert _{C^0} \, dr \\&\le 4 \Vert f\Vert _{C^0({\mathbb {R}}^d)} \Vert g\Vert _{L_q( (0, 1); C^\beta )} |t-s|^{1/q^*}, \end{aligned}$$

and notice that $1/q^*>1/2$ by (4.8). Moreover, by the regularity of *g*, we get

$$\begin{aligned} \Vert \textbf{E}^s \big ( \mathcal {A}_t&-\mathcal {A}_s- A_{s, t} \big ) \Vert _{L_p(\Omega )} \\&\big \Vert \int _s^t\big ( g(r,\bar{X}^n_r) - g(r,\bar{X}^n_s) \big ) \textbf{E}^s \big ( f(\bar{X}^n_r)-f({\bar{X}}^n_{\kappa _n(r)})\big )\, dr \big \Vert _{L_p(\Omega )} \\&\le 2 \Vert f\Vert _{C^0({\mathbb {R}}^d)} \int _s^t \Vert g(r, \cdot )\Vert _{C^\beta ({\mathbb {R}}^d)} |r-s|^{\beta /2} \, dr \\&\le 2 \Vert f\Vert _{C^0({\mathbb {R}}^d)} \Vert g\Vert _{L_q( (0, 1); C^\beta ({\mathbb {R}}^d))} |t-s|^{1/q^*+\beta /2}, \end{aligned}$$

and notice that $1/q^*+\beta /2>1$ from (4.8). Consequently, the stochastic sewing lemma (Lemma 4.1) gives

4.9 $$\begin{aligned}&\big \Vert \int _s^tg(r,\bar{X}^n_r) \big ( f(\bar{X}^n_r)-f(\bar{X}^n_{\kappa _n(r)})\big )\, dr \Vert _{L_p(\Omega )} = \Vert \mathcal {A}_t -\mathcal {A}_s\Vert _{L_p(\Omega )} \nonumber \\&\lesssim \Vert f\Vert _{C^0({\mathbb {R}}^d)} \Vert g\Vert _{L^q((0, 1);C^\beta ({\mathbb {R}}^d))} n^{-1/2+\gamma } |t-s|^{1/2} \nonumber \\&\lesssim \Vert g\Vert _{L^q((0, 1);C^\beta ({\mathbb {R}}^d))} ( \Vert f\Vert _{C^0({\mathbb {R}}^d)} + \Vert f\Vert _{L_\rho ({\mathbb {R}}^d)}) n^{-1/2+\gamma } |t-s| ^{1/2}, \end{aligned}$$

which gives the estimate in the case $f \in C^0({\mathbb {R}}^d) \cap L_\rho ({\mathbb {R}}^d)$.

*Step 2.* We continue with obtaining an estimate in the case $f \in C^0({\mathbb {R}}^d) \cap W_\rho ^k({\mathbb {R}}^d)$, where $k \in \mathbb {N}$ is such that $W^k_\rho ({\mathbb {R}}^d) \subset C^2({\mathbb {R}}^d)$. For |r-s|≥4/n, by writing Itô’s formula for $f(\bar{X}^n_r)$ is easy to see that

$$\begin{aligned} |\textbf{E}^s\big ( f(\bar{X}^n_r)-f({\bar{X}}^n_{\kappa _n(r)})\big )| \lesssim n^{-1} \Vert f\Vert _{C^2({\mathbb {R}}^d)}. \end{aligned}$$

On the other hand, if |r-s|≤4/n, then we have the trivial estimate

4.10 $$\begin{aligned} \Vert f(\bar{X}^n_r)-f({\bar{X}}^n_{\kappa _n(r)}) \Vert _{L_p(\Omega )} \le N \Vert f\Vert _{C^1({\mathbb {R}}^d)} n^{-1/2} \lesssim \Vert f\Vert _{C^1({\mathbb {R}}^d)} n^{-1+\gamma } |r-s|^{-1/2+\gamma }. \end{aligned}$$

Consequently, as before we get

$$\begin{aligned} \Vert A_{s, t}\Vert _{L_p(\Omega ) }&\lesssim \Vert g\Vert _{L^q((0, 1);C^\beta ({\mathbb {R}}^d))} \Vert f\Vert _{C^2({\mathbb {R}}^d)} n^{-1+\gamma } |t-s|^{(1+\gamma )/2}, \end{aligned}$$

for *q* sufficiently large. Moreover, similarly to the estimate (4.7), this time using (4.10) which is true for all *p*, and Hölder’s inequality, we arrive at

$$\begin{aligned} \Vert \textbf{E}^s \delta A_{s, u, t}\Vert _{L_p(\Omega )}&\lesssim \Vert g\Vert _{L^q((0, 1);C^\beta ({\mathbb {R}}^d))} \Vert f\Vert _{C^2({\mathbb {R}}^d)} n^{-1+\gamma } |t-s|^{-1/2+\gamma +1/q^*+\beta /2}, \end{aligned}$$

and as before, the stochastic sewing lemma (Lemma 4.1) guarantees that

$$\begin{aligned}&\qquad \big \Vert \int _s^tg(r,\bar{X}^n_r) \big ( f(\bar{X}^n_r)-f(\bar{X}^n_{\kappa _n(r)})\big )\, dr \Vert _{L_p(\Omega )} \\&\lesssim \Vert g\Vert _{L^q((0, 1);C^\beta ({\mathbb {R}}^d))} \Vert f\Vert _{C^2({\mathbb {R}}^d)} n^{-1+\gamma } |t-s|^{1/2} \\&\lesssim \Vert g\Vert _{L^q((0, 1);C^\beta ({\mathbb {R}}^d))} ( \Vert f\Vert _{C^0({\mathbb {R}}^d)} + \Vert f\Vert _{W^k_\rho ({\mathbb {R}}^d)}) n^{-1+\gamma } |t-s|^{1/2}, \end{aligned}$$

which gives us an estimate for the case $f \in C^0({\mathbb {R}}^d) \cap W^k_\rho ({\mathbb {R}}^d)$.

*Step 3.* By means of interpolation, the above bound and (4.9) show that for $f \in C^0({\mathbb {R}}^d) \cap W^\theta _\rho ({\mathbb {R}}^d)$, we have

$$\begin{aligned}&\big \Vert \int _s^tg(r,\bar{X}^n_r) \big ( f(\bar{X}^n_r)-f(\bar{X}^n_{\kappa _n(r)})\big )\, dr \Vert _{L_p(\Omega )} \\&\lesssim \Vert g\Vert _{L^q((0, 1);C^\beta ({\mathbb {R}}^d))} ( \Vert f\Vert _{C^0({\mathbb {R}}^d)} + \Vert f\Vert _{W^\theta _\rho ({\mathbb {R}}^d)}) n^{(-1+\gamma )\theta /k + (1-\theta /k)(-1/2+\gamma )} |t-s|^{1/2}. \\&= \Vert g\Vert _{L^q((0, 1);C^\beta ({\mathbb {R}}^d))} ( \Vert f\Vert _{C^0({\mathbb {R}}^d)} + \Vert f\Vert _{W^\theta _\rho ({\mathbb {R}}^d)}) n^{\gamma -1/2-\theta /(2k)} |t-s|^{1/2}. \end{aligned}$$

We can choose $\gamma < \theta /(2k)$ so that $\gamma -1/2-\theta /(2k)< -1/2$. The above estimate implies (4.5). □

### Lemma 4.3

Let $b \in C^0({\mathbb {R}}^d; {\mathbb {R}}^d)$, σ satisfy Assumption 2.1, p≥2, m≥1 and $f \in C^1({\mathbb {R}}^d)$. Suppose that *X*, $X^n$ satisfy (1.1) and (1.2), respectively. There exists $q_0 \ge 0$ and ε>0 such that for all $q \ge q_0$, $g \in L_q((0, 1); W^\alpha _m({\mathbb {R}}^d) \cap L_q({\mathbb {R}}^d) )$, $(s,t ) \in [0,1]_{\le }^2$, and $n\in \mathbb {N}$ one has the bound

$$\begin{aligned} \big \Vert \int _s^t g (r, X^n_r) \big ( f(X^n_r)-f(X^n_{\kappa _n(r)})\big )\, dr\big \Vert _{L_p(\Omega )}\le N n^{-(1+\varepsilon )/2}|t-s|^{1/2}, \end{aligned}$$

where *N* is a constant depending only on $\Vert g\Vert _{L_q((0, 1);W^\alpha _m \cap L_q)}, \Vert f\Vert _{C^1}, \Vert b\Vert _{C^0}, \Vert \sigma \Vert _{C^2}, p, d$, and λ.

### Proof

First, since *f* is Lipschitz, we have the trivial estimate

$$\begin{aligned}&\big \Vert \int _s^t g (r, X^n_r) \big ( f(X^n_r)-f(X^n_{\kappa _n(r)})\big )\, dr\big \Vert _{L_p(\Omega )}\\&\quad \lesssim \int _s^t \Vert g (r, X^n_r)\Vert _{L_{2p}(\Omega )} \Vert X^n_r - X^n_{\kappa _n(r)} \Vert _{L_{2p}(\Omega )} \, dr. \end{aligned}$$

Recall that the density of $X^n_r$ behaves similarly to the heat kernel at time *r* in Lebesgue spaces (see, e.g. Theorem 4.2 in [4]), which combined with the above estimate and the fact that $\Vert X^n_r - X^n_{\kappa _n(r)} \Vert _{L_{2p}(\Omega )} \le N n^{-1/2}$, shows that for all sufficiently large *q* we have

4.11 $$\begin{aligned}&\big \Vert \int _s^t g (r, X^n_r) \big ( f(X^n_r)-f(X^n_{\kappa _n(r)})\big )\, dr\big \Vert _{L_p(\Omega )}\nonumber \\&\quad \lesssim \Vert g\Vert _{L_q((0, 1) \times {\mathbb {R}}^d)} n^{-1/2} |t-s|^{1/2} \nonumber \\&\quad \lesssim \Vert g\Vert _{L_q((0, 1) ; L_q({\mathbb {R}}^d) \cap L_m({\mathbb {R}}^d))} n^{-1/2} |t-s|^{1/2}. \end{aligned}$$

On the other hand, suppose for now that $g \in L_q((0, 1) ; L_q({\mathbb {R}}^d) \cap W^k_m({\mathbb {R}}^d) )$ for *k* large so that $W^k_m({\mathbb {R}}^d) \subset C^1({\mathbb {R}}^d)$. Then, from Lemma 4.2, there exists ε>0 such that for *q* sufficiently large we have

$$\begin{aligned}&\big \Vert \int _s^t g (r, X^n_r) \big ( f(X^n_r)-f(X^n_{\kappa _n(r)})\big )\, dr\big \Vert _{L_p(\Omega )}\\&\quad \lesssim \Vert g\Vert _{L^q((0, 1);W^k_m({\mathbb {R}}^d) )} n^{-(1+\varepsilon )/2}|t-s|^{1/2} \\&\quad \lesssim \Vert g\Vert _{L^q((0, 1);L_q ({\mathbb {R}}^d) \cap W^k_m ({\mathbb {R}}^d))} n^{-(1+\varepsilon )/2}|t-s|^{1/2} . \end{aligned}$$

The result now follows by interpolation by means of the above bound and (4.11). □

## Proof of main results for Hölder *b*

### Hybrid Young–Itô differential equations

We establish some facts about the system (1.1),(1.4). We begin by proving Proposition 2.4.

#### Proof of Proposition 2.4

It clearly suffices to consider the operator $f \mapsto (Q^Xf)$ on $C^1({\mathbb {R}}^d)$, given by

$$ (Q^Xf)_t= \int _0^t \nabla f(X_s) \, ds, $$

and to show that on $C^1({\mathbb {R}}^d)$ it satisfies the estimate (iii). Moreover, by virtue of Girsanov’s theorem we may assume that the drift *b* is identically zero. To this end, let $z \in {\mathbb {R}}^d$ and let us set

$$\begin{aligned} \mathcal {A}_t= \int _0^t \big ( f (X_s)-f(X_s+z) \big ) \, ds, \qquad A_{s, t}= \textbf{E}^s \int _s^t \big ( f (X_r)-f(X_r+z) \big ) \, dr. \end{aligned}$$

Then, by the Markov property it follows that

$$\begin{aligned} A_{s, t}= \int _s^t \Big ( \mathcal {P}_{r-s}f(X_s)- \mathcal {P}_{r-s}f(X_s+z) \Big ) \, dr, \end{aligned}$$

where $(\mathcal {P}_t)_{t \in [0,1]}$ is the semigroup of the diffusion *X*. The following estimate is well-known (see, e.g., [15, Theorem 6.4.3])

$$\begin{aligned} | \mathcal {P}_tf(x)-\mathcal {P}_tf(y)| \lesssim \Vert f \Vert _{C^\alpha ({\mathbb {R}}^d)} |x-y| t^{(\alpha -1)/2}, \end{aligned}$$

which gives

$$\begin{aligned} \Vert A_{s, t} \Vert _{ L_p(\Omega )} \lesssim |z| \Vert f\Vert _{C^\alpha ({\mathbb {R}}^d)} |t-s|^{(1+\alpha )/2}. \end{aligned}$$

In addition, it is straightforward that $\textbf{E}^s \delta A_{s, u, t}=0$. It is also easy to see that the germs $(s, t) \rightarrow A_{s, t}$ reconstruct the process $t \rightarrow \mathcal {A}_t$ by means of the stochastic sewing lemma (Lemma 4.1). Hence we can conclude that

$$\begin{aligned} \big \Vert \int _s^t \big ( f (X_r)-f(X_r+z) \big ) \, dr \big \Vert _{L_p(\Omega )} \lesssim |z| \Vert f\Vert _{C^\alpha } |t-s|^{(1+\alpha )/2}. \end{aligned}$$

Since this was for arbitrary $z \in {\mathbb {R}}^d$, estimate (iii) follows and consequently the whole proposition is proved. □

The next result shows pathwise uniqueness for the system (1.1),(1.4).

#### Proposition 5.1

Let Assumptions 2.1 and 2.2 hold. Suppose that $$\{ (\Omega , \mathcal {F}, \mathbb {F}, \textbf{P}), X, V, B, W\}$$ and $$\{ (\Omega , \mathcal {F}, \mathbb {F}', \textbf{P}), X', V', B, W\}$$ are solutions of the system (1.1),(1.4). Then, we have that $X=X^{\prime }$ and $V=V^{\prime }$, $$\textbf{P}$$-almost surely.

#### Proof

The fact that $X=X^{\prime }$ is shown in [17]. Let us show that $V=V^{\prime }$. Recall that by definition, $$V, V' \in C^\beta ([0, 1]; {\mathbb {R}}^d )$$ with probability one, for some $$\beta \in ( (1-\alpha )/2, 1/2)$$. Recall also that by Remark 2.6, we can choose $$\alpha ' \in (0, (1+\alpha )/2)$$ such that $$L:= Q^Xb \in C^{\alpha '}([0, 1]; {\mathbb {R}}^{d \times d})$$ with probability one, and $$\alpha '+\beta >1$$. It is immediate that the difference $Y=V-V^{\prime }$ satisfies the equation

$$\begin{aligned} Y^{(\ell )}_t= \int _0^t Y^{(j)}_r dL^{(\ell , j)}_r+ \int _0^t Z^{(\ell , j, i)}_r Y^{(j)}_r \, dB^{(i)}_r, \qquad \ell \in \{1, \ldots , d \}, \end{aligned}$$

where $Z^{(\ell , j, i)}_\cdot = \partial _j \sigma ^{(\ell , i)}(X_\cdot )$. As usual, we drop the indices below for notational convenience. Next, let us denote by $\mathbb {G}=\{ \mathcal {G}_t \}_{ t \in [0, 1]}$, the augmentation of the filtration $\{ \sigma ( \mathcal {F}_s, \mathcal {F}'_s, s \le t ) \} _{t \in [0, 1]}$. Furthermore, let us choose $\tilde{\beta }<\beta< \tilde{\alpha } < \alpha '$ such that $\tilde{\alpha } + \tilde{\beta } >1$. Since $L \in C^{\alpha '}$, $Y \in C^{\beta }$, and $\tilde{\alpha }< \alpha '$, $\tilde{\beta } < \beta$, it follows that for any $\mathbb {G}$-stopping time ρ, the process $t \mapsto [L]_{C^{\tilde{ \alpha } }([\rho , \rho \vee t])}$ and $t \mapsto [Y]_{C^{\tilde{ \beta } }([\rho , \rho \vee t])}$ are Hölder continuous. In addition, they are $\mathbb {G}$-adapted. Let us take $\delta _1,\delta _2\in (0,1]$ (to be specified later) and define the stopping times $\rho _0=0$ and for $n\in \mathbb {N}$,

$$\begin{aligned} \rho _{n}:= \inf \{ t> \rho _{n-1} : [ L]_{C^{\tilde{\alpha }}([\rho _{n-1}, t])}>\delta _1 \text { or } t - \rho _{n-1}> \delta _2\}. \end{aligned}$$

Let us furthermore define

$$\begin{aligned} \tau := \inf \{ t> 0 : \Vert Y\Vert _{C^{\tilde{\beta }}([0, t])} > 1 \}\wedge 1 \end{aligned}$$

and set $\tau _n=\rho _n\wedge \tau$. Note that if $\Vert Y\Vert _{C^{\tilde{\beta }}([0,\tau ])}=0$, then τ=1 and Y≡0 on [0, 1].

Note that for any $n\in \mathbb {N}_0$ one has

5.1 $$\begin{aligned} [Y ]_{C^{\tilde{\beta }}([ \tau _n, \tau _{n+1}])} \le \Big [ \int _{\tau _n}^\cdot Y_r dL_r \Big ]_{C^{\tilde{\beta }}([ \tau _n, \tau _{n+1}])} + \Big [ \int _{\tau _n}^\cdot Z_r Y_r \, dB_r \Big ]_{C^{\tilde{\beta }}([ \tau _n, \tau _{n+1}])}. \end{aligned}$$

For the first term on the right hand side, we have with probability one

$$\begin{aligned} \Big [ \int _{\tau _n}^\cdot Y_r dL_r \Big ]_{C^{\tilde{\beta }}([ \tau _n, \tau _{n+1}])}&\le \Big [ \int _{\tau _n}^\cdot Y_r dL_r \Big ] _{C^{\tilde{\beta }}([ \tau _n, \tau _{n+1}])} \\&\le N \Vert Y \Vert _{C^{\tilde{\beta }}([ \tau _n, \tau _{n+1}])} [L]_{C^{\tilde{\alpha }}([ \tau _n, \tau _{n+1}])} \\&\le N\delta _1\big (|Y_{\tau _n}|+[Y]_{C^{\tilde{\beta }}([\tau _n,\tau _{n+1}])}\big ), \end{aligned}$$

where *N* depends only on $\tilde{\alpha }, \tilde{\beta }$ and *d*. For the second term, notice that

$$\begin{aligned} \Big [ \int _{\tau _n}^\cdot Z_r Y_r \, dB_r \Big ]_{C^\alpha ([ \tau _n, \tau _{n+1}])} = \Big [ \int _{0}^\cdot \textbf{1}_{ \tau _n \le r\le \tau _{n+1}} Z_r Y_r \, dB_r \Big ]_{C^\alpha ([0, 1])}. \end{aligned}$$

Hence, keeping in mind that $\tilde{\beta } <1/2$ and using Lemma A.2 and the Burkholder–Davis–Gundy (BDG) inequality, we have for all sufficiently large *p*

$$\begin{aligned} \Big [ \int _{\tau _n}^\cdot Z_r Y_r \, dB_r \Big ]_{L_p(\Omega ; C^{\tilde{\beta }}([ \tau _n, \tau _{n+1}]))}&\le N \Big [ \int _{0}^\cdot \textbf{1}_{ \tau _n \le r\le \tau _{n+1}} Z_r Y_r \, dB_r \Big ] _{C^{1/2} ([0, 1]; L_p(\Omega ))} \\&\le N \Vert Y \Vert _{L_p(\Omega ; L_\infty ([\tau _n, \tau _{n+1}]))} \\&\le N \Vert Y_{\tau _n} \Vert _{L_p(\Omega )}+ N \delta _2^{\tilde{\beta }} [ Y ]_{L_p(\Omega ; C^{\tilde{\beta }} ([ \tau _n, \tau _{n+1}]))}, \end{aligned}$$

where we have used that *Z* is a process bounded by $\Vert \sigma \Vert _{C^1}$, that by definition, $\tau _{n+1}-\tau _n \le \delta _2$, and *N* depends only on ${\tilde{\beta }}, p$, and *d*.

Therefore we can conclude that

$$ [ Y ]_{L_p(\Omega ; C^{\tilde{\beta }}([ \tau _n, \tau _{n+1}]))}\le N \Vert Y_{\tau _n} \Vert _{L_p(\Omega )} + N(\delta _1 + \delta _2^{\tilde{\beta }}) [ Y ]_{L_p(\Omega ; C^{\tilde{\beta }}([ \tau _n, \tau _{n+1}]))}, $$

where *N* does not depend on $\delta _1,\delta _2$. Upon assuming that $\delta _1$ and $\delta _2$ are sufficiently small, we get

$$ [ Y ]_{L_p(\Omega ; C^{\tilde{\beta }}([ \tau _n, \tau _{n+1}]))}\le N \Vert Y_{\tau _n} \Vert _{L_p(\Omega )}, $$

and by iteration this gives $[ Y ]_{L_p(\Omega ; C^{\tilde{\beta }}([ 0, \tau _{n+1}]))}=0$, for all $n \in \mathbb {N}$. For almost all $\omega \in \Omega$ we have that $\tau _n(\omega )=\tau (\omega )$ for all *n* large enough, which implies $[ Y ]_{L_p(\Omega ; C^{\tilde{\beta }}([ 0, \tau ]))}=0$, finishing the proof. □

The next proposition shows weak uniqueness for the system (1.1), (1.4). It follows from Proposition 5.1 and the result of Yamada and Watanabe [19, Proposition 1] (see also [11, Proposition 3.20, p. 309]).

#### Proposition 5.2

Let Assumptions 2.1 and 2.2 hold. Suppose that $$\{ (\Omega , \mathcal {F}, \mathbb {F}, \textbf{P}), X, V, B, W\}$$ and $$\{ (\Omega ', \mathcal {F}', \mathbb {F}', \textbf{P}'), X', V', B', W'\}$$ are solutions of the system (1.1), (1.4). Then the laws of (*X*, *V*, *B*, *W*) and $\left(X^{\prime },V^{\prime },B^{\prime },W^{\prime }\right)$ coincide.

### Uniform estimates and limiting processes

We obtain estimates for the processes $X^n,V^n$ which are uniform in n and establish the convergences to various processes which appear in equation (1.4).

#### Lemma 5.3

Let Assumptions 2.1 and 2.2 hold. Let $\{ (\Omega , \mathcal {F}, \mathbb {F}, \textbf{P}), X, B\}$ be a solution of (1.1) and define $X^n$ and $V^n$ be as in Theorem 2.9. Then, for any p≥2 and γ<1/2, we have

5.2 $$\begin{aligned} \sup _{n \in \mathbb {N}}\Vert V^n\Vert _{C^{1/2}([0, 1]; L_p(\Omega ))}< \infty , \qquad \sup _{n \in \mathbb {N}}\Vert V^n\Vert _{L_p(\Omega ; C^{\gamma }([0, 1]))} < \infty . \end{aligned}$$

#### Proof

Throughout the proof the proportionality constants in ≲ depend only on $d, \lambda , \alpha , \Vert b\Vert _{C^\alpha }, \Vert \sigma \Vert _{C^2}, p$.

We begin by proving the first inequality in (5.2). Let us set $Y^n=X-X^n$ and fix S,T∈[0,1] with S<T. For s,t∈[S,T] with s<t we have

5.3 $$\begin{aligned} Y^n_t-Y^n_s&= \int _s^t [b(X_r)- b(X^n_r)] \, dr + \int _s^t [b(X^n_r)- b(X^n_{\kappa _n (r)})] \, dr \nonumber \\&\quad +\int _s^t [\sigma (X_r)- \sigma (X^n_r)] \, d B_r+\int _s^t [\sigma (X^n_r)- \sigma (X^n_{\kappa _n(r)})] \, d B_r. \end{aligned}$$

For the first term in (5.3), by [1, Lemma 6.4] (with τ=1/2) we have that

$$\begin{aligned} \big \Vert \!\int _s^t [b(X_r)\!-\! b(X^n_r)] dr \big \Vert _{L_p(\Omega )}\! \lesssim \! |t-s| \Vert Y^n \Vert _{C^{1/2}([S, T]; L_p(\Omega ))} \!+\! |t-s|^{1/2}\Vert Y^n_S\Vert _{L_p(\Omega )}. \end{aligned}$$

For the second term in (5.3), by applying Lemma 4.2 with $\rho =\infty$, we get that

$$\begin{aligned} \big \Vert \int _s^t [b(X_r^n)-b(X_{\kappa _n(r)}^n)]\, dr\big \Vert _{L_p(\Omega )} \lesssim n^{-1/2-\varepsilon /2}|t-s|^{1/2} \end{aligned}$$

with some ε>0. For the third term, by the BDG inequality and the regularity of σ, one easily sees that

$$\begin{aligned}&\big \Vert \int _s^t [\sigma (X_r)- \sigma (X^n_r)] \, d B_r \big \Vert _{L_p(\Omega )} \lesssim |t-s|^{1/2} \sup _{r \in [s, t]} \Vert Y^n_r\Vert _{L_p(\Omega )} \\&\quad \lesssim |t-s|^{1/2}\big (|T-S|^{1/2} \Vert Y^n \Vert _{C^{1/2}([S, T]; L_p(\Omega ))} +\Vert Y^n_S\Vert _{L_p(\Omega )}\big ). \end{aligned}$$

Similarly, for the last term we have

$$\begin{aligned} \big \Vert \int _s^t [\sigma (X^n_r)- \sigma (X^n_{\kappa _n(r)})] \, d B_r \big \Vert _{L_p(\Omega )}&\lesssim |t-s|^{1/2} \sup _{r \in [s, t]} \Vert X^n_r-X^n_{\kappa _n(r)}\Vert _{L_p(\Omega )} \\&\lesssim |t-s|^{1/2} n^{-1/2}. \end{aligned}$$

Hence, combining all the above estimates with (5.3), gives

$$\begin{aligned} \Vert Y^n_t-Y^n_s\Vert _{L_p(\Omega )}\! \lesssim \! |t-s|^{1/2}\!\big (|T-S|^{1/2} \Vert Y^n \Vert _{C^{1/2}([S, T]; L_p(\Omega ))} \!+\!\Vert Y^n_S\Vert _{L_p(\Omega )}\!+\!n^{-1/2}\big ). \end{aligned}$$

Dividing by $|t-s|^{1/2}$ and taking suprema over s,t∈[S,T], gives

$$ [ Y^n] _{C^{1/2}([S, T]; L_p(\Omega ))} \lesssim \left( |T-S| ^{1/2} \Vert Y^n \Vert _{C^{1/2}([S, T]; L_p(\Omega ))} +\Vert Y^n_S\Vert _{L_p(\Omega )} + n^{-1/2} \right) , $$

which further gives

$$ \Vert Y^n \Vert _{C^{1/2}([S, T]; L_p(\Omega ))} \le N_0 \left( |T-S| ^{1/2} \Vert Y^n \Vert _{C^{1/2}([S, T]; L_p(\Omega ))} +\Vert Y^n_S\Vert _{L_p(\Omega )} + n^{-1/2} \right) $$

with $N_0$ not depending on *S*, *T*, *n*. If $|T-S| \le 1/(2N_0)$, the above gives

$$\begin{aligned} \Vert Y^n \Vert _{C^{1/2}([S, T]; L_p(\Omega ))} \lesssim \left( \Vert Y^n_S\Vert _{L_p(\Omega )} + n^{-1/2} \right) . \end{aligned}$$

Set now $S_k= k/ \lceil {2 N_0}\rceil$, $k =0, ..., \lceil {2 N_0}\rceil$. We obviously have $|S_{k+1}-S_k| \le 1/(2N_0)$, which gives

5.4 $$\begin{aligned} \Vert Y^n \Vert _{C^{1/2}([S_k, S_{k+1}]; L_p(\Omega ))} \lesssim \left( \Vert Y^n_{S_k}\Vert _{L_p(\Omega )} + n^{-1/2} \right) , \end{aligned}$$

for all $k =0, ..., \lceil {2 N_0}\rceil$. In particular, this gives

$$\begin{aligned} \Vert Y^n_{S_{k+1}}\Vert _{L_p(\Omega )} \lesssim \left( \Vert Y^n_{S_k}\Vert _{L_p(\Omega )} + n^{-1/2} \right) , \end{aligned}$$

which by iteration gives

$$\begin{aligned} \Vert Y^n_{S_k}\Vert _{L_p(\Omega )} \lesssim n^{-1/2}. \end{aligned}$$

Substituting the above into (5.4), we conclude that

$$\begin{aligned} \Vert Y^n \Vert _{C^{1/2}([S_k, S_{k+1}]; L_p(\Omega ))}\lesssim n^{-1/2} . \end{aligned}$$

Consequently, we get

$$\begin{aligned} \Vert Y^n \Vert _{C^{1/2}([0, 1]; L_p(\Omega ))}&\lesssim \sup _k \Vert Y^n \Vert _{C^{1/2}([S_k, S_{k+1}]; L_p(\Omega ))} \lesssim n^{-1/2}, \end{aligned}$$

which shows the first inequality of the claim. The second now follows from Lemma A.2. □

#### Lemma 5.4

Let Assumptions 2.1 and 2.2 hold. For $n \in \mathbb {N}$, let $\{ ( \bar{\Omega }, \bar{\mathcal {F}}, \bar{\textbf{P}}), \bar{\mathbb {F}}^n, \bar{\mathcal {X}}^n, \bar{B}^n\}$, $\{ ( \bar{\Omega }, \bar{\mathcal {F}}, \bar{\textbf{P}}), \bar{\mathbb {F}}, \bar{\mathcal {X}}, \bar{B}\}$, be solutions of (1.1). Define the processes

$$\begin{aligned} d\bar{X}^n_t&= b(\bar{X}^n_{\kappa _n(t)}) \, dt + \sigma (\bar{X}^n_{\kappa _n(t)}) \, d\bar{B}^n_t, \qquad \bar{X}^n_0=x_0,\\ \bar{V}^n&= \sqrt{n}( \bar{\mathcal {X}}^n- \bar{X}^n). \end{aligned}$$

Finally, let $\beta \in (0, 1/2]$, $q, \bar{q}\ge 1$ such that $(1+\alpha )/2+\beta >1$ and ${\bar{q}}<q$, and assume that

(i) There exists ${\bar{V}} \in L_q({\bar{\Omega }} ; C^\beta ([0, 1]; {\mathbb {R}}^d))$ such that $lim_{n \rightarrow \infty } \Vert {\bar{V}}\!-\!{\bar{V}}^n\Vert _{ L_q({\bar{\Omega }}; C^\beta ([0,1])) }=0$,
(ii) $$lim_{n \rightarrow \infty } \sup _{t \in [0, 1]} \Vert \bar{\mathcal {X}}^n_t -\bar{\mathcal {X}}_t\Vert _{ L_{q{\bar{q}}/(q-{\bar{q}})}({\bar{\Omega }})}=0$$.

Then, for any $f \in C^{\alpha +}({\mathbb {R}}^d)$ and $\gamma \in (0, \alpha )$ we have

5.5 $$\begin{aligned} \lim _{n \rightarrow \infty } \Big \Vert \int _0^\cdot \sqrt{n} (f(\bar{\mathcal {X}}^n_r)-f({\bar{X}}^n_r))\, dr - \int _0^ \cdot {\bar{V}}^{(j)}_r \, d( Q^{\bar{\mathcal {X}}}f)^{(j)}_r \Big \Vert _{C^{(1+\gamma )/2} ([0, 1] ; L_{{\bar{q}}}( {\bar{\Omega }}))} =0 . \end{aligned}$$

#### Proof

Let $\varepsilon \in (0,1)$ be small so that

$$\begin{aligned} \theta +\beta := (1+\alpha )/2-\varepsilon + \beta >1,\qquad (1+\gamma )/2 < \theta . \end{aligned}$$

Throughout the proof the proportionality constants in ≲ depend only on $\alpha ,\beta ,\gamma ,\varepsilon ,d,q,{\bar{q}},$ and $\Vert {\bar{V}}\Vert _{L_q({\bar{\Omega }};C^\beta ([0,1]))}$. Notice that by Lemma 2.5, for $f\in C^{\alpha +}$ we have that $Q^{\bar{\mathcal {X}}}f \in L_p ( {\bar{\Omega }} ; C^\theta ([0,1]; {\mathbb {R}}^d))$ for all p≥1. In particular, the Young integrals appearing in (5.5) are well defined and satisfy

$$\begin{aligned} \big \Vert \int _s^t \bar{V}^{(j)}_r \, d ( Q^{\bar{\mathcal {X}}}f)^{(j)}_r \big \Vert _{L_{\bar{q}}({\bar{\Omega }})}&\lesssim \big \Vert \Vert \bar{V} \Vert _{C^\beta ([0,1])} \Vert Q^{\bar{\mathcal {X}}}f \Vert _{C^\theta ([0,1])} \big \Vert _{L_{\bar{q}}({\bar{\Omega }})} |t-s|^\theta \\&\lesssim \Vert \bar{V} \Vert _{L_q({\bar{\Omega }} ; C^\beta ([0,1]) ) } \Vert Q^{\bar{\mathcal {X}}}f \Vert _{L_{q{\bar{q}}/(q-{\bar{q}})}( {\bar{\Omega }}; C^{ \theta }([0,1]) ) } |t-s|^\theta \\&\lesssim \Vert f\Vert _{C^{\alpha }({\mathbb {R}}^d)} |t-s|^\theta , \end{aligned}$$

where we have used again Remark 2.5. Hence, the mapping $\mathcal {I}$ given by

$$\begin{aligned} f \mapsto \sum _{j=1}^d \int _0^\cdot \bar{V}^{(j)}_r \, d ( Q^{\bar{\mathcal {X}}}f)^{(j)}_r \end{aligned}$$

is a bounded linear operator from $C^{\alpha +} ( {\mathbb {R}}^d)$ to $C^\theta ( [0,1]; L_{{\bar{q}}} ({\bar{\Omega }}))$. Let us similarly define the operators $\mathcal {I}^n:C^{\alpha +} ( {\mathbb {R}}^d) \rightarrow C^\theta ( [0,1]; L_{{\bar{q}}} ({\bar{\Omega }}))$ by

$$\begin{aligned} f \mapsto \int _0^\cdot \sqrt{n} (f(\bar{ \mathcal {X}}^n_r)-f({\bar{X}}^n_r))\, dr. \end{aligned}$$

By applying [1, Lemma 6.4] with $\tau =\beta$ we get

$$\begin{aligned} \big \Vert \int _s^t \sqrt{n} (f(\bar{ \mathcal {X}}^n_r)-f({\bar{X}}^n_r))\, dr \big \Vert _{L_{{\bar{q}}}({\bar{\Omega }})} \lesssim \Vert V^n \Vert _{ C^\beta ([0, 1]; L_{{\bar{q}}}({\bar{\Omega }}) ) } \Vert f \Vert _{C^{\alpha }({\mathbb {R}}^d)} |t-s|^\theta . \end{aligned}$$

By assumption (*i*) and the trivial embedding $L_{{\bar{q}}}({\bar{\Omega }}; C^\beta ([0, 1]))\subset C^\beta ([0, 1]; L_{{\bar{q}}}({\bar{\Omega }}) )$ we get that

5.6 $$\begin{aligned} \sup _{n\in \mathbb {N}} \Vert \mathcal {I}^n\Vert _{\mathcal {L}(C^{\alpha +} ( {\mathbb {R}}^d); C^\theta ( [0,1]; L_{{\bar{q}}} ({\bar{\Omega }}))}\lesssim 1. \end{aligned}$$

If $g \in C^\infty ({\mathbb {R}}^d)$, by Proposition 2.4 (i) and the fundamental theorem of calculus we have

$$\begin{aligned} \Vert&(\mathcal {I}^n_t(g)- \mathcal {I}^n_s(g))-( \mathcal {I}_t(g)- \mathcal {I}_s(g))\Vert _{L_{{\bar{q}}}({\bar{\Omega }})} \\&=\ \big \Vert \int _s^t \big ( \int _0^1 \nabla g (\bar{\mathcal {X}}^n_r + \vartheta ( {\bar{X}}^n_r -\bar{\mathcal {X}}^n_r) ) {\bar{V}}^n_r \, d \vartheta - \nabla g(\bar{\mathcal {X}}_r) {\bar{V}}_r \big ) \, dr \big \Vert _{L_{{\bar{q}}}({\bar{\Omega }})} \\&\le \Vert g\Vert _{C^2}|t-s| \Big ( \sup _{t \in [0, 1]} \Vert {\bar{V}}^n_t-{\bar{V}}_t \Vert _{L_{{\bar{q}}}({\bar{\Omega }})}+ \sup _{t \in [0, 1]} \Vert (\bar{\mathcal {X}}^n_t-\bar{\mathcal {X}}_t) {\bar{V}}_t \Vert _{L_{{\bar{q}}}({\bar{\Omega }})} \\&\quad + \sup _{t \in [0, 1]} \Vert ({\bar{X}}^n_t-\bar{\mathcal {X}}^n_t) {\bar{V}}_t \Vert _{L_{{\bar{q}}}({\bar{\Omega }})} \Big ) \\&\le \Vert g\Vert _{C^2}|t-s| \Big ( \Vert V-V^n\Vert _{ L_q({\bar{\Omega }}; C^\beta ([0,1])) } \\&\quad + \big ( \sup _{t \in [0, 1]} \Vert (\bar{\mathcal {X}}^n_t-\bar{\mathcal {X}}_t) \Vert _{L_{q{\bar{q}}/(q-{\bar{q}})}({\bar{\Omega }})} \\&\quad + \sup _{t \in [0, 1]} \Vert {\bar{X}}^n_t-\bar{\mathcal {X}}^n_t \Vert _{L_{q{\bar{q}}/(q-{\bar{q}})}({\bar{\Omega }})} \big ) \Vert V\Vert _{C^\beta ([0,1]; L_q({\bar{\Omega }}) )} \Big ). \end{aligned}$$

From (*i*) and (*ii*) of our assumptions combined with [1, Theorem 2.1], we conclude that for $g \in C^\infty ({\mathbb {R}}^d)$

$$\begin{aligned} \lim _{n \rightarrow \infty } \Vert \mathcal {I}_n(g)- \mathcal {I}(g)\Vert _{C^\theta ([0, 1] ; L_{{\bar{q}}}( {\bar{\Omega }}))} =0. \end{aligned}$$

Since $f \in C^{\alpha +}({\mathbb {R}}^d)$, for any δ>0, we can find $\tilde{f} \ \in C^\infty ({\mathbb {R}}^d)$ such that $\Vert f -\tilde{f} \Vert _{C^{\alpha }({\mathbb {R}}^d)}\le \delta$. Then, we see that

$$\begin{aligned}&\Vert \mathcal {I}_n(f)- \mathcal {I}(f)\Vert _{C^\theta ([0, 1] ; L_{{\bar{q}}}( {\bar{\Omega }}))} \\  &\le \Vert \mathcal {I}_n(f)- \mathcal {I}_n(\tilde{f})\Vert _{C^\theta ([0, 1] ; L_{{\bar{q}}}( {\bar{\Omega }}))} + \Vert \mathcal {I}_n(\tilde{f})- \mathcal {I}(\tilde{f})\Vert _{C^\theta ([0, 1] ; L_{{\bar{q}}}( {\bar{\Omega }}))} \\&\quad + \Vert \mathcal {I}(f)- \mathcal {I}(\tilde{f})\Vert _{C^\theta ([0, 1] ; L_{{\bar{q}}}( {\bar{\Omega }}))} \\&\le ( \Vert \mathcal {I}_n\Vert + \Vert \mathcal {I}\Vert ) \Vert f -\tilde{f} \Vert _{C^{\alpha }({\mathbb {R}}^d)}+ \Vert \mathcal {I}_n(\tilde{f})- \mathcal {I}(\tilde{f})\Vert _{C^\theta ([0, 1] ; L_{{\bar{q}}}( {\bar{\Omega }}))}, \end{aligned}$$

which implies that $\limsup _{n \rightarrow \infty }\Vert \mathcal {I}_n(f)- \mathcal {I}(f)\Vert _{C^\theta ([0, 1] ; L_{{\bar{q}}}( {\bar{\Omega }}))} \lesssim \delta$, and since δ>0 was arbitrary and $\theta > (1+\gamma )/2$, the claim is proved. □

We will make use of the following proposition which is well known, see for instance [6, Theorem 5.1].

#### Proposition 5.5

Let $(\Omega , \mathcal {F}, \textbf{P})$ be a probability space carrying an ${\mathbb {R}}^d$-valued Brownian motion *B* and define $W^n$ as in Theorem 2.9. There exists an extension $(\tilde{\Omega }, \tilde{\mathcal {F}}, \tilde{\textbf{P}})$ of $(\Omega , \mathcal {F}, \textbf{P})$, carrying a ${\mathbb {R}}^{d \times d}$-valued standard Brownian motion *W* independent of *B*, such that the laws of $(W^n,B)$ converge weakly to the law of (*W*, *B*) on $C([0, 1];{\mathbb {R}}^d\times {\mathbb {R}}^{d \times d})$.

### Proof of Theorems 2.8 and 2.9

#### Proof of Theorems 2.8 and 2.9

The uniqueness part of Theorem 2.8 follows from Proposition 5.2. Let us set $S^n=(X, V^n, B, W^n)$. We start by showing that any subsequence $\{S^n\}_{n \in \mathbb {N}_1} \subset \{S^n\}_{n \in \mathbb {N}}$, there exists a further subsequence $\{S^n\}_{n \in \mathbb {N}_2} \subset \{S^n\}_{n \in \mathbb {N}_1}$ which converges weakly to a solution of the system (1.1), (1.4). This will verify the existence part of Theorem 2.8 and since $\{S^n\}_{n \in \mathbb {N}_1}$ is arbitrary, will also prove Theorem 2.9.

*Step 1.* Let $\{S^n\}_{n \in \mathbb {N}_1}$ be an arbitrary subsequence. We show that there is a further subsequence $\{(X, X^n, V^n, B, W^n)\}_{n \in \mathbb {N}_2}$ that converges weakly (on suitable metric spaces) to a limit denoted by $(\hat{ \mathcal {X}}, \hat{X}, \hat{V}, \hat{B}, {\hat{W}})$ and furthermore that $\hat{ \mathcal {X}}= \hat{X}$.

Let us fix $\beta ,\gamma ,\gamma ' \in (0, 1/2)$ such that $\gamma> \gamma '> \beta >(1-\alpha )/2$. Moreover, we choose q>1 sufficiently large such that

5.7 $$\begin{aligned} C^{\gamma }([0,1]) \subset \subset W^{\gamma '}_q ([0, 1]) \subset \subset C^\beta ([0,1]). \end{aligned}$$

For any p≥1, by Lemma 5.3, we have that

5.8 $$\begin{aligned} \sup _{n \in \mathbb {N}_1} \Vert V^n\Vert _{L_p(\Omega , C^\gamma ([0,1]; {\mathbb {R}}^d))} < \infty . \end{aligned}$$

Moreover, by the boundedness of the coefficients and the BDG inequality one can easily see that $(X, X^n, B, W^n)$ is bounded in $L_p(\Omega ; \mathcal {C}^\gamma )$, where

$$\begin{aligned} \mathcal {C}^\gamma = \big ( C^\gamma ([0, 1]; {\mathbb {R}}^d) \big )^3 \times C^\gamma ([0, 1]; {\mathbb {R}}^{d\times d}). \end{aligned}$$

By the first compact embedding in (5.7), we have that the laws of $\{(X, X^n, V^n, B, W^n)\}_{n \in \mathbb {N}_1}$ are tight on

$$\begin{aligned} \mathcal {W}^{\gamma '}_q=\big (W^{\gamma '}_q ([0, 1]; {\mathbb {R}}^d ) \big )^4 \times W^{\gamma '}_q ([0, 1]; {\mathbb {R}}^{d \times d} ) . \end{aligned}$$

The latter is a Polish space and, therefore, by Prokorov’s theorem, we have that the laws of a further subsequence $\{(X, X^n, V^n, B, W^n)\}_{n \in \mathbb {N}_2}$ converge weakly. By Skorohod’s representation theorem, there exists a probability space $( \hat{\Omega }, \hat{\mathcal {F}}, \hat{\textbf{P}})$ and random variables

$$\begin{aligned} (\hat{ \mathcal {X}}^{n}, \hat{X}^n, \hat{V}^n, \hat{B}^n , {\hat{W}}^n) : \hat{\Omega } \rightarrow \mathcal {W}^{\gamma '}_q, \quad n \in \mathbb {N}_2, \end{aligned}$$

and

$$\begin{aligned} (\hat{ \mathcal {X}}, \hat{X}, \hat{V}, \hat{B}, {\hat{W}}) : \hat{\Omega } \rightarrow \mathcal {W}^{\gamma '}_q, \end{aligned}$$

such that

5.9 $$\begin{aligned} (\hat{ \mathcal {X}}^{n}, \hat{X}^n, \hat{V}^n, \hat{B}^n , {\hat{W}}^n) \overset{d}{=}\ (X, X^n, V^n, B, W^n) \ \text { on } \ \mathcal {W}^{\gamma '}_q, \end{aligned}$$

and

5.10 $$\begin{aligned} \hat{\textbf{P}}\text {-a.s. }\lim _{n\rightarrow \infty }(\hat{ \mathcal {X}}^{n}, {\hat{X}}^n, {\hat{V}}^n, {\hat{B}}^n , {\hat{W}}^n) = (\hat{ \mathcal {X}}, {\hat{X}}, {\hat{V}}, {\hat{B}}, {\hat{W}}) \ \text { in }\ \mathcal {W}^{\gamma '}_q. \end{aligned}$$

Let $\{{\hat{\mathcal {F}}}^n_t\}_{t \ge 0}$ be the augmentation of the filtration generated by $\hat{ \mathcal {X}}^n, {\hat{B}}^n$, and ${\hat{W}}^n$. It is easy to check that ${\hat{B}}^n$ is a $\{{\hat{\mathcal {F}}}^n_t\}_{t \ge 0}$-Brownian motion. Notice that by (5.9) and the continuity of the coefficients, the following equalities follow

5.11 $$\begin{aligned} d \hat{ \mathcal {X}}^n_t&= b( \hat{ \mathcal {X}}^n_t) \, dt + \sigma ( \hat{ \mathcal {X}}^n_t) \, d {\hat{B}}^n_t, \qquad X^{(n)}_0=x_0 , \end{aligned}$$

5.12 $$\begin{aligned} d {\hat{X}}^n_t&= b( {\hat{X}}^n_{\kappa _n(t)} ) \, dt + \sigma ( {\hat{X}}^n_{\kappa _n(t)} ) \, d {\hat{B}}^n_t, \qquad X^{(n)}_0=x_0 , \end{aligned}$$

5.13 $$\begin{aligned} {\hat{V}}^n&= \sqrt{n}(\hat{ \mathcal {X}}^n_t- {\hat{X}}^n_t ) , \end{aligned}$$

5.14 $$\begin{aligned} {\hat{W}}^n_t&= \sqrt{2 n} \int _0^t ( {\hat{B}}^n_r -{\hat{B}}^n_{\kappa _n(r)} ) \, d {\hat{B}}_r. \end{aligned}$$

By (5.9) and the fact that $$ (X, X^n, V^n, B, W^n)$$ are bounded in $$L_p(\Omega ; \mathcal {W}^{\gamma '}_q)$$ for any p≥1, we get that

5.15 $$\begin{aligned} \sup _{n \in \mathbb {N}_2}\Vert (\hat{ \mathcal {X}}^{n}, {\hat{X}}^n, {\hat{V}}^n, {\hat{B}}^n , {\hat{W}}^n)\Vert _{L_p({\hat{\Omega }}; \mathcal {W}^{\gamma '}_q)}< \infty , \end{aligned}$$

for any p≥1. The almost sure convergence from (5.10) combined with the uniform integrability obtained by (5.15) gives by Vitali’s theorem that for all p≥1

5.16 $$\begin{aligned} \lim _{n \rightarrow \infty }\Vert (\hat{ \mathcal {X}}^{n}, {\hat{X}}^n, {\hat{V}}^n, {\hat{B}}^n , {\hat{W}}^n)- (\hat{ \mathcal {X}}, {\hat{X}}, {\hat{V}}, {\hat{B}}, {\hat{W}})\Vert _{L_p({\hat{\Omega }}; \mathcal {W}^{\gamma '}_q)}=0. \end{aligned}$$

Next, we show that $\hat{ \mathcal {X}} =\hat{X}.$ By (5.16) we have that $\hat{ \mathcal {X}}^n \rightarrow \hat{ \mathcal {X}}$ and $\hat{X}^n \rightarrow \hat{X}$ while (5.11)-(5.12) and [1, Theorem 2.7] imply that $\hat{ \mathcal {X}}^n -\hat{X}^n \rightarrow 0$, where all three limits are understood, for example, in $C([0,1];{\mathbb {R}}^d)$ in probability (recall the second embedding in (5.7)).

*Step 2.* Let $\hat{\mathbb {F}}:= \{{\hat{\mathcal {F}}}_t\}_{t \in [0, 1]}$ be the augmentation of the filtration generated by $\hat{X}, \hat{V}, {\hat{B}}, {\hat{W}}$. To conclude the proofs, we show that $\{ ( {\hat{\Omega }}, {\hat{\mathcal {F}}}, \hat{\mathbb {F}}, {\hat{\textbf{P}}}), \hat{ X}, {\hat{V}}, {\hat{B}}, {\hat{W}} \}$ is a solution of the system (1.1), (1.4).

In view of (5.9), (5.10), and Proposition 5.5, it follows that ${\hat{B}}$ and ${\hat{W}}$ are independent Brownian motions, that ${\hat{B}}$ is an $\hat{\mathbb {F}}$-Brownian motion, and that ${\hat{W}}$ is an $\hat{\mathbb {F}}$-martingale. This suffices to conclude that ${\hat{W}}$ is also an $\hat{\mathbb {F}}$-Brownian motion. By the convergence in (5.10) combined with the continuity of the coefficients and Lemma A.1, we can let $n \rightarrow \infty$ in (5.11) to obtain that $\{(\hat{\Omega }, \hat{\mathcal {F}}, \hat{\mathbb {F}}, \hat{\textbf{P}}), \hat{ \mathcal {X}}, {\hat{B}}\}$ is a solution of (1.1). Moreover, since pathwise uniqueness holds for (1.1), it follows that $\mathcal {X}$ is adapted to the augmentation of the filtration generated by $\hat{B}$. In particular, this shows that $( \mathcal {X}, {\hat{B}}) \perp \!\!\! \perp \hat{W}$, since ${\hat{B}} \perp \!\!\! \perp \hat{W}$ . Consequently, (i) and (ii) from Definition 2.7 are satisfied. Moreover, (iii) follows from the fact that ${\hat{V}} \in W^{\gamma '}_q ([0, 1]; {\mathbb {R}}^d)$ and the second embedding in (5.7).

Finally, we focus on showing (iv), that is, showing that ${\hat{V}}=({\hat{V}}^{(1)},\ldots ,{\hat{V}}^{(d)})$ satisfies the equation

5.17 $$\begin{aligned} d {\hat{V}}_t= d ( Q^Xb)_t {\hat{V}} _t + \nabla \sigma ( {\hat{X}}_r) {\hat{V}}_rd {\hat{B}}_r+\frac{1}{\sqrt{2}}\nabla \sigma \sigma ( {\hat{X}}_r)d {\hat{W}}_r. \end{aligned}$$

From (5.11)-(5.12), we have

5.18 $$\begin{aligned} {\hat{V}}^n_t&= \sqrt{n} \int _0^t \big ( b (\hat{ \mathcal {X}}^{n}_s)- b ( {\hat{X}}^n_{\kappa _n(s)}) \big ) \, ds + \sqrt{n} \int _0^t \big ( \sigma (\hat{ \mathcal {X}}^{n}_s)- \sigma ( {\hat{X}}^n_{\kappa _n(s)}) \big ) \, d {\hat{B}}^n_s \nonumber \\&=I^n_t+E^n_t+{\bar{I}}^n_t+{\bar{E}}^n_t, \end{aligned}$$

where

$$\begin{aligned} I^n_t&=\int _0^t \sqrt{n}(b( \hat{ \mathcal {X}}^n_r)-b ({\hat{X}}^n_r))dr, \\ E^n_t&=\int _0^t\sqrt{n}( b({\hat{X}}^n_r)-b ({\hat{X}}^n_{\kappa _n(r)}))dr, \\ {\bar{I}}^n_t&= \int _0^t\sqrt{n} (\sigma ( \hat{ \mathcal {X}}^n_r)-\sigma ({\hat{X}}^n_r))d{\hat{B}}^n_r, \\ {\bar{E}}^{n}_t&=\int _0^t\sqrt{n}(\sigma ({\hat{X}}^n_r)-\sigma ({\hat{X}}^n_{\kappa _n(r)}))d {\hat{B}}^n_r. \end{aligned}$$

By (5.16) and (5.7), we see that the conditions of Lemma 5.4 are satisfied, and Lemma 5.4 combined with Lemma A.2 implies that

5.19 $$\begin{aligned} \big \Vert I^n- \int _0^ \cdot \, d (Q^{{\hat{X}}}b)_r {\hat{V}}_r\big \Vert _{C^\beta ([0,1])} \xrightarrow {\hat{\textbf{P}}} 0, \end{aligned}$$

as $n \rightarrow \infty$. Here and below $\xrightarrow {\hat{\textbf{P}}}$ denotes convergence in probability. In addition, by Lemma 4.2 and Lemma A.2 we have

5.20 $$\begin{aligned} \Vert E^{n}\Vert _{C^\beta ([0,1])} \xrightarrow {\hat{\textbf{P}}} 0, \end{aligned}$$

as $n \rightarrow \infty$. Moving to ${\bar{I}}^n$, by the fundamental theorem of calculus we have that

$$\begin{aligned} {\bar{I}}^n_t&= \int _0^t\sqrt{n} (\sigma ( \hat{ \mathcal {X}}^n_r)-\sigma ({\hat{X}}^n_r))d{\hat{B}}^n_r \\&= \int _0^t \int _0^1 \nabla \sigma ( \hat{ \mathcal {X}}^n_r+ \vartheta ( {\hat{X}}^n_r-\hat{ \mathcal {X}}^n_r)) \, d \vartheta \, \hat{V}^n_r \,d{\hat{B}}^n_r. \end{aligned}$$

By (5.16) and the fact that $\sigma \in C^2$, we get that

$$\begin{aligned} \big \Vert \int _0^1 \nabla \sigma ( \hat{ \mathcal {X}}^n + \vartheta ( {\hat{X}}^n-\hat{ \mathcal {X}}^n)) \, d \vartheta \, \hat{V}^n - \nabla \sigma (\hat{X} )\, {\hat{V}} \big \Vert _{C([0,1])} \xrightarrow {\hat{\textbf{P}}} 0, \end{aligned}$$

and

$$\begin{aligned} \Vert {\hat{B}}^n-{\hat{B}}\Vert _{C([0,1])} \xrightarrow {\hat{\textbf{P}}} 0, \end{aligned}$$

as $n \rightarrow \infty$. Consequently, by Lemma A.1, we get that

5.21 $$\begin{aligned} \big \Vert {\bar{I}}^n - \int _0^ \cdot \nabla \sigma (\hat{X}_r ) {\hat{V}}_r\, d \hat{B}_r\Vert _{C([0,1])} \xrightarrow {\hat{\textbf{P}}} 0, \end{aligned}$$

as $n \rightarrow \infty$. Finally, we deal with ${\bar{E}}^n$. By the fundamental theorem of calculus again, we have

$$\begin{aligned} {\bar{E}}^n_t&= \int _0^t \int _0^1 \nabla \sigma \big ( \hat{X}^n_r+ \vartheta ( \hat{X}^n_{\kappa _n(r)}- \hat{X}^n_r) \big ) \, d \vartheta \sqrt{n} ( \hat{X}^n_r- \hat{X}^n_{\kappa _n(r)} ) \, d {\hat{B}}^n_r \\&= \int _0^t \int _0^1 \nabla \sigma \big ( \hat{X}^n_r+ \vartheta ( \hat{X}^n_{\kappa _n(r)}- \hat{X}^n_r) \big ) \, d \vartheta \sqrt{n} (r-\kappa _n(r)) b( \hat{X}^n_{\kappa _n(r)}) \, d {\hat{B}}^n_r \\&\qquad + \frac{1}{\sqrt{2}} \int _0^t \int _0^1 \nabla \sigma \big ( \hat{X}^n_r+ \vartheta ( \hat{X}^n_{\kappa _n(r)}- \hat{X}^n_r) \big ) \, d \vartheta \,\sigma ( \hat{X}^n_{\kappa _n(r)}) d \hat{W}^n_r \\&=: {\bar{E}}^{n, 1}_t + {\bar{E}}^{n, 2}_t. \end{aligned}$$

Since $(r-\kappa _n(r))$ is of order $n^{-1}$ and since *b* and $\nabla \sigma$ are bounded, it follows by Davis’ inequality that $\Vert {\bar{E}}^{n, 1} \Vert _{C([0,1])} \xrightarrow {\hat{\textbf{P}}} 0$. Next, by (5.16) it follows that we have

$$\begin{aligned} \sup _{r \in [0,1]}| \int _0^1 \nabla \sigma \big ( \hat{X}^n_r+ \vartheta ( \hat{X}^n_{\kappa _n(r)}- \hat{X}^n_r) \big ) \, d \vartheta \sigma ( \hat{X}^n_{\kappa _n(r)}) - \nabla \sigma ( \hat{X}_r)\sigma (\hat{X}_r)| \xrightarrow {\hat{\textbf{P}}} 0, \end{aligned}$$

and

$$\begin{aligned} \Vert {\hat{W}}^n-{\hat{W}} \Vert _{C([0,1])} \xrightarrow {\hat{\textbf{P}}} 0, \end{aligned}$$

as $n \rightarrow \infty$. Consequently, by Lemma A.1, we get that

5.22 $$\begin{aligned} \big \Vert {\bar{E}}^n -\frac{1}{\sqrt{2}}\int _0^ \cdot \nabla \sigma ( \hat{X}_r)\sigma (\hat{X}_r) \, d \hat{W}_r\Vert _{C([0,1])} \xrightarrow {\hat{\textbf{P}}} 0, \end{aligned}$$

as $n \rightarrow \infty$. Recall that almost surely $\Vert \hat{V}^n-\hat{V}\Vert _{C([0,1])}\rightarrow 0$, which combined with (5.19)-(5.22), upon letting $n \rightarrow \infty$ in (5.18) leads to (5.17). Hence, this shows that (iv) from Definition 2.7 is also satisfied. This finishes the proof. □

## Proof of main results for Sobolev *b*

### Tightness

In this section we focus on the following tightness result.

#### Theorem 6.1

Let Assumptions 2.1 and 3.1 hold. For any p≥1 and $\gamma \in (0, 1)$ we have

$$\begin{aligned} \sup _{n\in \mathbb {N}} \Vert V^n \Vert _{C^{\gamma /2}([0, 1]; L_p(\Omega ))} < \infty . \end{aligned}$$

In particular, for any $\gamma \in (0, 1)$, the laws of $\{V^n\}_{n \in \mathbb {N}}$ are tight on $C^{\gamma /2}([0, 1])$.

Before proceeding to the proof of the above theorem, we need some auxiliary results. Let *u* be the solution of (1.7), for a θ>0 to be determined later on. By applying Itô’s formula for $u(t, X_t)$, we obtain that

6.1 $$\begin{aligned} \int _0^t b(X_r) d r \!=\! u(0,X_0)-u(t, X_t) \!+\! \theta \int _0^t u(r, X_r) d r \!+\! \int _0^t (\partial _i u \sigma ^{(i,j)})(r, X_r) d B^{(j)}_r, \end{aligned}$$

and similarly,

6.2 $$\begin{aligned} \int _0^t b(X^n_r) d r&= u(0,X^n_0)-u(t, X^n_t) + \theta \int _0^t u(r, X^n_r) d r\nonumber \\&\quad +\int _0^t \partial _i u(r, X_r^n) [b^{(i)}( X_{\kappa _n(r)}^n)-b^{(i)}(X^n_r)] d r \nonumber \\&\quad +\int _0^t \partial _{ij}^2 u(r,X^n_r)[a^{(i,j)}(X^n_{\kappa _n(r)})-a^{(i,j)}(X^n_r)]dr \nonumber \\&\quad +\int _0^t \partial _i u(r,X^n_r)\sigma ^{(i,j)}(X^n_{\kappa _n(r)})dB^{(j)}_r. \end{aligned}$$

From equations (1.1) and (1.2), we have

$$\begin{aligned} X_t-X^n_t&=x_0-x_0^n +\int _0^t[b(X_r)-b(X^n_r)]dr +\int _0^t[b(X^n_r)-b(X^n_{\kappa _n(r)})]dr\\&\quad +\int _0^t[\sigma (X_r)-\sigma (X^n_r)]dB_r +\int _0^t[\sigma (X^n_r)-\sigma (X^n_{\kappa _n(r)})]dB_r. \end{aligned}$$

We plug (6.1) and (6.2) into the identity above to see that

6.3 $$\begin{aligned} X_t-X^n_t =x_0-x^n_0+\sum _{k=0}^6 J^k_{t} \end{aligned}$$

where for each $\ell \in \{1, \ldots , d\}$

6.4 $$\begin{aligned} \begin{aligned} J^{0,(\ell )}_t&=u^{(\ell )}(t,X_t)-u^{(\ell )}(t,X^n_t) ,\\J^{1,(\ell )}_t&=\theta \int _0^t (u^{(\ell )}(r,X_r)-u^{(\ell )}(r,X^n_r))dr ,\\J^{2,(\ell )}_t&=\int _0^t[I^{(\ell ,i)}+\partial _i u^{(\ell )}(r,X^n_r)][b^{(i)}(X^n_r)-b^{(i)}(X^n_{\kappa _n(r)})]dr ,\\J^{3,(\ell )}_t&=\int _0^t \partial _{i j}^2 u^{(\ell )}(r,X^n_r)[a^{(i,j)}(X^n_r)-a^{(i,j)}(X^n_{\kappa _n(r)})]dr ,\\J^{4,(\ell )}_t&=\int _0^t[I^{(\ell ,i)}+\partial _i u^{(\ell )}(r,X^n_r)][\sigma ^{(i,j)}(X_r)-\sigma ^{(i,j)}(X^n_r)]dB^{(j)}_r ,\\J^{5,(\ell )}_t&=\int _0^t[I^{(\ell ,i)}+\partial _i u^{(\ell )}(r,X^n_r)][\sigma ^{(i,j)}(X^n_r)-\sigma ^{(i,j)}(X^n_{\kappa _n(r)})]dB^{(j)}_r ,\\J^{6,(\ell )}_t&=\int _0^t[\partial _i u^{(\ell )}(r,X_r)-\partial _i u^{(\ell )}(r,X^n_r)] \sigma ^{(i,j)}(X_r)dB^{(j)}_r. \end{aligned} \end{aligned}$$

Finally, let us introduce the process

6.5 $$\begin{aligned} A_t^n:= t+\int _0^t \left[ \mathcal {M}|\nabla ^2 u|(s,X_s)+\mathcal {M}|\nabla ^2 u|(s,X^n_s)\right] ^2 d s, \end{aligned}$$

where $\mathcal {M}$ is the Hardy–Littlewood maximal operator (in the spatial variable) defined as

$$\begin{aligned} \mathcal {M}f(x):=\sup _{0<r<\infty }\frac{1}{|\mathcal {B}_r|}\int _{\mathcal {B}_r}f(x+y)dy, \quad \mathcal {B}_r:=\{x\in \mathbb {R}^d:|x|<r\},\quad r>0. \end{aligned}$$

It is well known that there exists a constant *N* depedning only on *d* such that for all $f \in W^1_{1, loc}({\mathbb {R}}^d)$, for almost every $x, y \in {\mathbb {R}}^d$, we have

6.6 $$\begin{aligned} |f(x)-f(y)| \le N |x-y|\big ( \mathcal {M}|\nabla f| (x) + \mathcal {M}|\nabla f| (y) \big ), \end{aligned}$$

and that for all $f \in L_p({\mathbb {R}}^d)$, $p \in (1, \infty )$, we have

6.7 $$\begin{aligned} \Vert \mathcal {M}f \Vert _{L_p({\mathbb {R}}^d)} \le N \Vert f \Vert _{L_p( R^d)}. \end{aligned}$$

The following is a straightforward consequence of the boundedness of the coefficients and the BDG inequality.

#### Lemma 6.2

Let $X^n$ be defined by (1.2) and assume that *b* and σ are bounded. Then for every $p \in [1, \infty )$, there exists a constant $N=N(p, \Vert b\Vert _{C^0}, \Vert \sigma \Vert _{C^2})$, such that for all $n \in \mathbb {N}$ we have

$$\begin{aligned} \sup _{t\in [0,1]}\Vert X^n_{t}-X^n_{\kappa _n(t)}\Vert _{L_p(\Omega )}\le N n^{-1/2}. \end{aligned}$$

For the proof of Proposition 6.4 below, we will need the following Krylov-type estimate.

#### Lemma 6.3

Suppose that Assumption 2.1 holds and $b \in C^0({\mathbb {R}}^d; {\mathbb {R}}^d)$ and let p>(d+2)/2. Let $X^n$ be defined by (1.2). There exists a constant $N=(\Vert b\Vert _{C^0}, \Vert \sigma \Vert _{C^2}, \lambda , d,p)$ such that for all $g \in L_p((0, 1) \times {\mathbb {R}}^d)$ and all $(s, t) \in [0, 1]_{\le }$ we have

$$\begin{aligned} \textbf{E}\int _s^t g(r,X^n_r) \, dr \le N (t-s)^{1-(d+2)/(2p)} \Vert g\Vert _{L_p((0, 1) \times {\mathbb {R}}^d)}. \end{aligned}$$

#### Proof

It suffices to prove the claim for *g* bounded with compact support. Let $\bar{X}^n$ be the Euler scheme corresponding to the driftless equation. From [14, Lemma 5.5] we have that

6.8 $$\begin{aligned} \textbf{E}f(\bar{X}^n_r) \lesssim r^{-d/2q} \Vert f\Vert _{L_q({\mathbb {R}}^d)}, \end{aligned}$$

for any function *f* and any $q \in [1, \infty )$. Let us also denote by $\rho _n$ the Girsanov density, that is

$$\begin{aligned} \rho _n= \exp \Big ( - \int _0^1 b \sigma ^{-1} (X^n_{\kappa _n(s)}) dB_s-\frac{1}{2}\int _0^1 b \sigma ^{-1} (X^n_{\kappa _n(s)}) dB_s \Big ), \end{aligned}$$

and notice that due to the boundedness of *b* and the uniform allipticity of σ, $\rho _n$ and $\rho _n^{-1}$ have finite moments of any order uniformly in $n\in \mathbb {N}$. Then, by Hölder’s inequality and (6.8) with q=2, $f= |g|^{p/2}$, we have

$$\begin{aligned}&\textbf{E}|g(r, X^n_r)| = \textbf{E}|g(r, X^n_r)| \rho ^{2/p} \rho ^{-2/p}\\&\quad \lesssim \big ( \textbf{E}|g(r, X^n_r)|^{p/2} \rho \big )^{2/p}= \big ( \textbf{E}|g(r, \bar{X}^n_r)|^{p/2} \big )^{2/p}\\&\quad \lesssim \big (r^{-d/4} \Vert g(r, \cdot ) \Vert _{L_p({\mathbb {R}}^d)}^{p/2} \big )^{2/p} = r^{-d/2p} \Vert g(r, \cdot ) \Vert _{L_p({\mathbb {R}}^d)}. \end{aligned}$$

Hence, integrating the above inequality from *s* to *t* and applying Hölder’s inequality proves the claim.

#### Proposition 6.4

Let Assumptions 2.1 and 3.1 hold, and let *X* and $X^n$ satisfy (1.1) and (1.2), respectively. For every $p\in [1,\infty )$, there exists a constant $N=N(p, d, \Vert b\Vert _{C^0}, \Vert b\Vert _{W^\alpha _m}, \Vert \sigma \Vert _{C^2})$, such that for all $n \in \mathbb {N}$ we have

6.9 $$\begin{aligned} \big \Vert \sup _{t\in [0,1]}|X_t-X^n_t|\big \Vert _{L_{p}(\Omega )}\le N n^{-1/2}. \end{aligned}$$

#### Proof

The argument is similar to [14, Section 7], however, the rate in (6.9) is improved under Assumption 3.1. The details are presented below.

We raise (6.3) to *p*-th power to find that

$$\begin{aligned} \xi _t:=\sup _{s\in [0,t]}|X_s-X^n_s|^p \lesssim |x_0-x_0^n|^p+\sum _{i=0}^6\sup _{s\in [0,t]}|J^i_s|^p. \end{aligned}$$

Using (3.2) and Hölder’s inequality, we get

$$\begin{aligned} \sup _{s\in [0,t]}(| J^0_s|^p+| J^1_s|^p) \lesssim \theta ^{-p/2}\sup _{s\in [0,t]}|X_s-X_s^n|^p+ \theta ^{p/2}\left( \int _0^t \xi _r^{2/p} dr\right) ^{\frac{p}{2}}. \end{aligned}$$

To estimate $J^4,J^5,J^6$, we will utilise a special case of the pathwise BDG inequality of [16, Theorem 5]. Namely, there exists a constant C=C(p,d) such that for any cádlág martingale ${\bar{M}}$, there exists a local martingale ${\hat{M}}$ such that with probability one,

$$\begin{aligned} \sup _{s\in [0,t]}|{\bar{M}}_s|^p\le C[{\bar{M}}]_t^{p/2}+{\hat{M}}_t, \quad \forall t. \end{aligned}$$

In the above, $[{\bar{M}}]$ is the quadratic variation of ${\bar{M}}$. Using the Lipschitz regularity of σ, (3.2) and the pathwise BDG inequality, we can find a local martingale $M^4$ such that

$$\begin{aligned} \sup _{s\in [0,t]}| J^4_s|^p&\lesssim \left( \int _0^t|X_r-X^n_r|^2dr\right) ^{\frac{p}{2}}+M^4_t\\&\le \!\left( \int _0^t \xi _r^{2/p}dA^n_r \right) ^{\frac{p}{2}}+M^4_t. \end{aligned}$$

By (6.6), for almost every r∈[0,1] and almost every $x,y\in {\mathbb {R}}^d$, we have

$$\begin{aligned} |\nabla u(r,x)- \nabla u(r,y)|\lesssim |x-y|(\mathcal {M}|\nabla ^2u|(r,x)+\mathcal {M}|\nabla ^2u|(r,y)). \end{aligned}$$

Applying this (keeping in mind that the laws of $X_r$ and $X^n_r$ are absolutely continuous) and the pathwise BDG inequality, we get

$$\begin{aligned} \sup _{s\in [0,t]}| J^6_s|^p&\lesssim \left( \int _0^t|X_r-X^n_r|^2(\mathcal {M}|\nabla ^2u|(r,X_r)+\mathcal {M}|\nabla ^2u|(r,X^n_r))^2dr\right) ^{\frac{p}{2}}+M^6_t\\&\le \left( \int _0^t \xi _r^{2/p}dA^n_r \right) ^{\frac{p}{2}}+M^6_t \end{aligned}$$

for some local martingale $M^6$. Using Lemma 6.2, (3.2), Lipschitz regularity of σ and the pathwise BDG inequality, we have

$$\begin{aligned} \sup _{s\in [0,t]}|J^5_s|^p\lesssim \left( \int _0^t|X^n_r-X^n_{\kappa _n(r)}|^{2}dr\right) ^{\frac{p}{2}}+M^5_t. \end{aligned}$$

It follows that

$$\begin{aligned} \xi _t\lesssim \theta ^{-1/2} \xi _t+ (1+\theta ^{p/2})\left( \int _0^t \xi ^{2/p}dA^n\right) ^{\frac{p}{2}}+J^*_t+M_t, \end{aligned}$$

where

$$ J^*_t=|x_0-x^n_0|^p+\sum _{i=2}^3\sup _{s\in [0,t]}|J^i_s|^p+\left( \int _0^t|X^n_r-X^n_{\kappa _n(r)}|^{2}dr\right) ^{\frac{p}{2}} $$

and $M=M^4+M^5+M^6$. By choosing θ sufficiently large, this deduces to

$$\begin{aligned} \xi _t\lesssim \left( \int _0^t \xi ^{2/p}dA^n\right) ^{\frac{p}{2}}+J^*_t+M_t. \end{aligned}$$

Applying stochastic Grönwall lemma ( [14, Lemma 3.8]), we have

6.10 $$\begin{aligned} \textbf{E}e^{-c_p |A^n_1|^{\max (p/2,1)}} \xi _1\lesssim \textbf{E}J^*_1. \end{aligned}$$

We proceed with an estimate for the right-hand-side of the above inequality. Notice that by Sobolev embedding and (3.1), we have that $\nabla u \in L_{q}((0, 1); C^{1-\delta }({\mathbb {R}}^d))$ for any $\delta \in (0, 1)$ and q≥1. By applying Lemma 4.2 with the choice g=I+∇u and f=b, and Lemma A.2, we have

$$\begin{aligned} \textbf{E}\sup _{s\in [0,1]}| J^2_s|^p \lesssim n^{-p/2}. \end{aligned}$$

Using the Lipschitz continuity and the boundedeness of σ, by the Cauchy–Schwarz inequality, we have

$$\begin{aligned} \textbf{E}\sup _{s\in [0,1]}| J^3_s|^p&\lesssim \textbf{E}\Big |\int _0^1|\nabla ^2 u(r,X^n_r)||X^n_r-X^n_{\kappa _n(r)}| dr \Big |^p\\&\le \textbf{E}\left( \int _0^t|\nabla ^2 u(r,X^n_r)|^2dr\right) ^{\frac{p}{2}}\left( \int _0^1|X^n_r-X^n_{\kappa _n(r)}|^2 dr \right) ^{\frac{p}{2}}\\&\le \! \left[ \textbf{E}\left( \int _0^1|\nabla ^2 u(r,X^n_r)|^2dr\right) ^{p}\right] ^{1/2}\! \left[ \textbf{E}\left( \int _0^1|X^n_r\!-\!X^n_{\kappa _n(r)}|^{2} dr \right) ^{p}\right] ^{1/2}. \end{aligned}$$

By Jensen’s inequality, the Krylov-type estimate from Lemma 6.3 and (3.1) we see that the first factor on the right-hand side above is bounded uniformly in *n*. Hence, applying Lemma 6.2, we obtain that

$$\begin{aligned} \textbf{E}\sup _{s\in [0,1]}|J^3_s|^p\lesssim n^{-p/2}. \end{aligned}$$

The previous estimates for $\textbf{E}\sup _{s\in [0,t]}|J^i_s|^p$, i=2,3 combined with Lemma 6.2 and (6.10) imply that

$$\begin{aligned} \big \Vert e^{-c_p|A^n_1|^{\max (p/2,1)}}\sup _{t\in [0,1]}|X_t-X^n_t|\big \Vert _{L_p(\Omega )}\lesssim n^{-1/2}. \end{aligned}$$

Under Assumptions 2.1 and 3.1, it follows from [14, Lemma 7.4] that $\sup _n\textbf{E}e^{\kappa |A^n_1|^\rho }$ is finite for every κ>0 and ρ>0. In addition, by Hölder’s inequality,

$$\begin{aligned} \big \Vert \sup _{t\in [0,1]}|X_t-X^n_t|\big \Vert _{L_p(\Omega )}\le \big \Vert e^{c_{2p}|A^n_1|^p}\big \Vert _{L_{2p}(\Omega )}^{1/2} \big \Vert e^{-c_{2 p}|A^n_1|^p}\sup _{t\in [0,1]}|X_t-X^n_t|\big \Vert _{L_{2p}(\Omega )}^{1/2} . \end{aligned}$$

Combining with the previous estimates, we obtain (6.9).

#### Proof of Theorem 6.1

Define $Z^n_t=X_t-X^n_t$. From (6.3), we have

6.11 $$\begin{aligned} \Vert Z^n_{s,t}\Vert _{L_p(\Omega )}\le \sum _{i=0}^6 \Vert J^i_{s,t}\Vert _{L_p(\Omega )}. \end{aligned}$$

Here and below we use the shorthand $f_{s,t}=f_t-f_s$. By the fundamental theorem of calculus we have

$$\begin{aligned} J^0_{s, t}&= \int _0^1 \nabla u(t, X^n_t+ \theta (X_t-X^n_t)) Z^n_{s, t} \, d\theta \\&\qquad + \int _0^1 \big (\nabla u(t, X^n_t+ \theta (X_t-X^n_t))-\nabla u(s, X^n_s+ \theta (X_s-X^n_s)) \big ) Z^n_s \, d\theta . \end{aligned}$$

Let us fix $\gamma \in (0, 1)$. By using (3.2) and (3.3) we get

$$\begin{aligned} \Vert J^0_{s, t} \Vert _{L_p(\Omega )} \lesssim \theta ^{-1/2} \Vert Z^n_{s, t} \Vert _{L_p(\Omega )} + \Vert (|t-s|^{\gamma /2} +| X^n_{s, t}|^{\gamma }+|X_{s, t}|^{\gamma }) |Z^n_{s}| \Vert _{L_p(\Omega )}\\ \lesssim \theta ^{-1/2} \Vert Z^n_{s, t} \Vert _{L_p(\Omega )} + |t-s|^{\gamma /2}n^{-1/2}, \end{aligned}$$

where for the last step we have used Hölder’s inequality and Theorem 6.4. We estimate the remaining terms at the right hand side of (6.11) as in the proof of Theorem 6.4, however this time, taking also into account (6.9). From (3.2) and (6.9),

$$\begin{aligned} \Vert J^1_{s,t}\Vert _{L_p(\Omega )}\lesssim \theta ^{1/2}\int _s^t \Vert X_r-X^n_r\Vert _{L_p(\Omega )}dr\lesssim \theta ^{1/2} n^{-1/2}(t-s) . \end{aligned}$$

Next, notice that by Sobolev embedding and (3.1), we have that $\nabla u \in L_q((0, 1); C^{1-\delta }({\mathbb {R}}^d))$ for any $\delta \in (0, 1)$ and q≥1. By applying Lemma 4.2 with the choice g=I+∇u and f=b, we have that

6.12 $$\begin{aligned} \Vert J^2_{s,t}\Vert _{L_p(\Omega )} \le |t-s|^{1/2} n^{-1/2-\varepsilon }. \end{aligned}$$

By (3.4) it follows that $\nabla ^2u$ satisfies the conditions of *g* in Lemma 4.3, hence we get

6.13 $$\begin{aligned} \Vert J^3_{s,t}\Vert _{L_p(\Omega )}\lesssim n^{-1/2-\varepsilon }(t-s)^{1/2}. \end{aligned}$$

By the BDG inequality, (3.3), the Lipschitz continuity of σ and Proposition Proposition 6.4, we get

$$\begin{aligned} \Vert J^4_{s,t}\Vert _{L_p(\Omega )}\lesssim n^{-1/2} |t-s|^{1/2}. \end{aligned}$$

Similarly, by using Lemma 6.2 in place of Proposition 6.4, we get

$$\begin{aligned} \Vert J^5_{s,t}\Vert _{L_p(\Omega )}\lesssim \left\| \left( \int _s^t|X^n_r-X^n_{\kappa _n(r)}|^2dr\right) ^{1/2} \right\| _{L_p(\Omega )}\lesssim n^{-1/2} |t-s|^{1/2}. \end{aligned}$$

By the BDG inequality, (6.9), Hölder’s inequality, and Proposition 6.4, we get

$$\begin{aligned} \Vert J^6_{s,t}\Vert _{L_p(\Omega )}\lesssim \Vert \sup _{r\in [0,1]}|X_r-X^n_r| | A^n_{s,t}|^{1/2} \Vert _{L_p(\Omega )} \lesssim n^{-1/2} \Vert A^n_{s,t} \Vert _{L_p(\Omega )}^{1/2}. \end{aligned}$$

Then, by Hölder’s inequality, Krylov’s estimate and its variant Lemma 6.3, we get

$$\begin{aligned} \Vert A^n_{s,t} \Vert ^p_{L_p(\Omega )}\!&\lesssim \!|t-s|^{p-1}\! \textbf{E}\int _s^t |\mathcal {M} |\nabla ^2 u | (r, X_r) |^{2p} \!+\! |\mathcal {M} |\nabla ^2 u |(r, X^n_r) |^{2p} \, dr \!+\!|t-s|^p\\&\lesssim |t-s|^{p-1+1-(d+2)/(2q)} ( \Vert \nabla ^2u \Vert ^{2p}_{L_{2p q}((0, 1) \times {\mathbb {R}}^d)}+1), \end{aligned}$$

provided that q>(d+2)/2. Hence, for *q* large enough, the above combined with (3.1) gives

$$\begin{aligned} \Vert A^n_{s,t} \Vert ^p_{L_p(\Omega )}&\lesssim |t-s|^{\gamma p}, \end{aligned}$$

which implies that

$$\begin{aligned} \Vert J^6_{s,t}\Vert _{L_p(\Omega )}\lesssim |t-s|^{\gamma /2} n^{-1/2}. \end{aligned}$$

Combining all these estimates, gives, with some constant *N* independent of θ and *n*,

$$\begin{aligned} \Vert Z^n_{s, t} \Vert _{L_p(\Omega )} \le N( \theta ^{-1/2} \Vert Z^n_{s, t} \Vert _{L_p(\Omega )} + |t-s|^{\gamma /2}n^{-1/2} ). \end{aligned}$$

Hence, provided that θ is sufficiently large, the result follows.

### Proof of Theorems 3.5 and 3.6

#### Proof of Theorems 3.5 and 3.6

We adopt a similar proof strategy as in Section 5. First, we show that the system (1.1),(1.9) has a unique solution. Next, with the usual notation, let us set $S^n=(X, V^n, B, W^n)$. We show that for any subsequence $\{S^n\}_{n \in \mathbb {N}_1} \subset \{S^n\}_{n \in \mathbb {N}}$, there exists a further subsequence $\{S^n\}_{n \in \mathbb {N}_2} \subset \{S^n\}_{n \in \mathbb {N}_1}$ which converges weakly to a solution of the system (1.1),(1.9). Clearly, because of the uniqueness part, this shows the existence part of Theorem 3.5 as well as the weak convergence result of Theorem 3.6.

*Step 1.* We show the uniqueness part of Theorem 3.5.

Recall that the matrix I-∇u(t,x) is invertible and both I-∇u(t,x) and $(I- \nabla u (t, x))^{-1}$ are bounded uniformly in (*t*, *x*). Then, it is easy to see that if (*X*, *V*) satisfies (1.1),(1.9), then $Z:=\big (I-\nabla u(\cdot , X_\cdot )\big )V$ satisfies the linear (Itô) equation

6.14 $$\begin{aligned} Z_t&= \theta \int _0^t\nabla u(r,X_r) \big (I-\nabla u(r, X_r) \big )^{-1} Z_r\, dr\nonumber \\&\quad +\int _0^t[\nabla \big ( (\nabla u + I ) \sigma \big ) ](r,X_r)\big (I-\nabla u(r, X_r) \big )^{-1} Z_rdB_r\nonumber \\&\quad +\frac{1}{\sqrt{2}}\int _0^t(I+\nabla u)\nabla \sigma \sigma (r,X_r) dW_r. \end{aligned}$$

For the system (1.1),(6.14) we have that strong uniqueness holds, and by the result of Yamada and Watanabe [19, Proposition 1] (see also [11, Proposition 3.20, p. 309]) uniqueness in law holds as well. Consequently, if $$\{ (\Omega , \mathcal {F}, \mathbb {F}, \textbf{P}), X, V, B, W\}$$ and $$\{ (\Omega ', \mathcal {F}', \mathbb {F}', \textbf{P}'), X', V', B', W'\}$$ are solutions of the system (1.1),(1.9), then the laws of $$(X, (I-\nabla u (\cdot , X_{\cdot })) V, B, W)$$ and $$(X', (I-\nabla u (\cdot , X'_{\cdot })) V', B', W')$$ on $$\mathcal {C}$$ coincide. Since the map $$(x, y) \mapsto (x, (I-\nabla u(t, x)) y) $$ is a measurable bijection for each t≥0, it follows that the finite dimensional distributions of (*X*, *V*, *B*, *W*) and $\left(X^{\prime },V^{\prime },B^{\prime },W^{\prime }\right)$ coincide, hence, so does their distributions on $$\mathcal {C}$$.

*Step 2.* Let $\{S^n\}_{n \in \mathbb {N}_1}$ be an arbitrary subsequence. We fix $\beta \in (0, 1/2)$, and by arguing as in the proof of Theorems 2.8 and 2.9, this time using the compactness result from Theorem 6.1, we have that the exists a probability space $( {\hat{\Omega }}, {\hat{\mathcal {F}}}, {\hat{\textbf{P}}})$ and random variables

$$\begin{aligned} (\hat{ \mathcal {X}}^{n}, {\hat{X}}^n, {\hat{V}}^n, {\hat{B}}^n , {\hat{W}}^n) : {\hat{\Omega }} \rightarrow \mathcal {C}^{\beta }=: \big (C^\beta ([0, 1] ; {\mathbb {R}}^d)^4 \times \big ) \times C^\beta ([0, 1] ; {\mathbb {R}}^{d\times d}) \end{aligned}$$

for $$n \in \mathbb {N}_2$$, and

$$\begin{aligned} (\hat{ \mathcal {X}}, {\hat{X}}, {\hat{V}}, {\hat{B}}, {\hat{W}}) : {\hat{\Omega }} \rightarrow \mathcal {C}^{\beta }, \end{aligned}$$

such that

6.15 $$\begin{aligned} (\hat{ \mathcal {X}}^{n}, {\hat{X}}^n, {\hat{V}}^n, {\hat{B}}^n , {\hat{W}}^n) \overset{d}{=}\ (X, X^n, V^n, B, W^n) \ \text { on } \ \mathcal {C}^{\beta }, \end{aligned}$$

and

6.16 $$\begin{aligned} (\hat{ \mathcal {X}}^{n}, {\hat{X}}^n, {\hat{V}}^n, {\hat{B}}^n , {\hat{W}}^n) \rightarrow (\hat{ \mathcal {X}}, {\hat{X}}, {\hat{V}}, {\hat{B}}, {\hat{W}}) \ \text { as } \ n \rightarrow \infty , \end{aligned}$$

${\hat{\textbf{P}}}$-a.s. in $\mathcal {C}^{\beta }$ and also in $L_p(\hat{\Omega }; \mathcal {C}^\beta )$. By (6.15), (6.16), and Proposition 6.4 it follows that $\hat{\mathcal {X}}=\hat{X}$. Therefore, we have

6.17 $$\begin{aligned} (\hat{ \mathcal {X}}^{n}, {\hat{X}}^n, {\hat{V}}^n, {\hat{B}}^n , {\hat{W}}^n) \rightarrow (\hat{X}, {\hat{X}}, {\hat{V}}, {\hat{B}}, {\hat{W}}) \ \text { as } \ n \rightarrow \infty , \end{aligned}$$

${\hat{\textbf{P}}}$-a.s. in $\mathcal {C}^{\beta }$ and also in $L_p(\hat{\Omega }; \mathcal {C}^\beta )$. Let $\{{\hat{\mathcal {F}}}^n_t\}_{t \ge 0}$ be the augmentation of the filtration generated by $\hat{ \mathcal {X}}^n, {\hat{B}}^n$, and ${\hat{W}}^n$. It follows again that ${\hat{B}}^n$ is a $\{{\hat{\mathcal {F}}}^n_t\}_{t \ge 0}$-Brownian motion and that the equalities (5.12), (5.13), and (5.14), are satisfied.

We claim that (5.11) is also satisfied. Indeed, let us fix t∈[0,1] and let π be a partition on [0, *t*]. Let $b_m$ be a mollification of *b*, and let us set

$$\begin{aligned}&\hat{\mathbb {S}}_{\pi }= x_0+ \sum _{[s, s' ] \in \pi } \sigma ( \hat{\mathcal {X}}^n_s) \hat{B}^n_{s, s'}, \ \ \hat{\mathbb {D}}_{\pi }\\&\quad = \sum _{[s, s' ] \in \pi } b( \hat{\mathcal {X}}^n_s) (s'-s), \ \ \hat{\mathbb {D}}^m_{\pi }= \sum _{[s, s' ] \in \pi } b_m( \hat{\mathcal {X}}^n_s) (s'-s), \end{aligned}$$

and

$$\begin{aligned} \hat{\mathbb {S}}= x_0+ \int _0^ t \sigma ( \hat{\mathcal {X}}^n_s) \, d\hat{B}^n_s , \ \ \hat{\mathbb {D}}= \int _0^t b( \hat{\mathcal {X}}^n_s) \, ds , \ \ \hat{\mathbb {D}}^m= \int _0^t b_m( \hat{\mathcal {X}}^n_s) \, ds , \end{aligned}$$

We also define $\mathbb {S}_{\pi }, \mathbb {D}_{\pi },\mathbb {D}^m _{\pi },\mathbb {S}, \mathbb {D}$ and, $\mathbb {D}^m$, similarly but with $\hat{\mathcal {X}}^n$, $\hat{B}^n$ replaced by *X*, *B*. By (6.15) it follows that the distributions of $(\hat{\mathcal {X}}^n, \hat{B}^n)$ and (*X*, *B*) coincide, which in particular implies that $(\hat{\mathcal {X}}^n_t,\hat{\mathbb {S}}_{\pi }, \hat{\mathbb {D}}^m_{\pi })$ and $(X_t,\mathbb {S}_{\pi }, \mathbb {D}^m_{\pi })$ have the same distribution. Using the continuity of $b_m$ and σ, we can let let $|\pi | \rightarrow 0$, and conclude that $(\hat{\mathcal {X}}^n_t,\hat{\mathbb {S}}, \hat{\mathbb {D}}^m)$ and $(X_t,\mathbb {S}, \mathbb {D}^m)$ have the same distribution. Next, we want to let $m \rightarrow \infty$, in order to conclude that $(\hat{\mathcal {X}}^n_t,\hat{\mathbb {S}}, \hat{\mathbb {D}})$ and $(X_t,\mathbb {S}, \mathbb {D})$ have the same distribution. For this, it suffices to show that $\mathbb {D}^m \rightarrow \mathbb {D}$ and $\hat{\mathbb {D}}^m \rightarrow \hat{\mathbb {D}}$ in probability. Notice that since $b_m \rightarrow b$ almost everywhere and since the law of $X_s$ is absolutely continuous for any s∈[0,1], we can use the boundedness of *b* in order to get from Lebesgue’s theorem on dominated convergence that $\mathbb {D}^m \rightarrow \mathbb {D}$ in $L_1(\Omega )$, hence also in probability. Similarly we get that $\hat{\mathbb {D}}^m \rightarrow \hat{\mathbb {D}}$ in probability. Consequently, the laws of $(\hat{\mathcal {X}}^n_t,\hat{\mathbb {S}}, \hat{\mathbb {D}})$ and $(\mathcal {X}^n_t,\mathbb {S}, \mathbb {D})$ coincide. In particular, this means that $(\hat{\mathcal {X}}^n, \hat{B}^n)$ satisfies (5.11).

*Step 3.* Let $\hat{\mathbb {F}}:= \{{\hat{\mathcal {F}}}_t\}_{t \in [0, 1]}$ be the augmentation of the filtration generated by $\hat{X}, V, {\hat{B}}, {\hat{W}}$. We verify that $\{ ( {\hat{\Omega }}, {\hat{\mathcal {F}}}, \hat{\mathbb {F}}, {\hat{\textbf{P}}}), \hat{ X}, {\hat{V}}, {\hat{B}}, {\hat{W}} \}$ is a solution of the system (1.1),(1.9).

The fact that ${\hat{B}}$ and ${\hat{W}}$ are independent $\hat{\mathbb {F}}$-Brownian motions with values in ${\mathbb {R}}^d$ and $\mathbb {R}^{d\times d}$, respectively, is shown in the proof of Theorems 2.8 and 2.9. To show that $\{(\hat{\Omega }, \hat{\mathcal {F}}, \hat{\mathbb {F}}, \hat{\textbf{P}}), \hat{ \mathcal {X}}, {\hat{B}}\}$ is a solution of (1.1), notice that since the distributions of $(\hat{\mathcal {X}}^n, \hat{B}^n)$ and (*X*, *B*) coincide for all $n\in \mathbb {N}$, we also get that the distributions of $(\hat{\mathcal {X}}, \hat{B})$ and (*X*, *B*) also coincide. From here, we can repeat the above argument where we showed that $(\hat{\mathcal {X}}^n, \hat{B}^n)$ satisfies (5.11) in order to obtain that $(\hat{\mathcal {X}}, \hat{B})$ solves (1.1). Moreover, since pathwise uniqueness holds for (1.1), it follows that $\mathcal {X}$ is adapted to the augmentation of the filtration generated by $\hat{B}$. In particular, this shows that $( \mathcal {X}, {\hat{B}}) \perp \!\!\! \perp \hat{W}$, since ${\hat{B}} \perp \!\!\! \perp \hat{W}$ . Consequently, (i) and (ii) from Definition 3.4 are satisfied. Moreover, (iii) follows from the construction of $\hat{\mathbb {F}}$ and the fact that with probability one $V \in C^\beta ([0, 1])$.

We focus on showing (iv). Let $u=(u^{(1)},\ldots ,u^{(d)})$, where $u^{(\ell )}$, for $\ell \in \{1, ..., d\}$ is the solution to (1.7). Using Itô’s formula, we have

6.18 $$\begin{aligned} \hat{V}^n_t=\sqrt{n}\sum _{i=0}^6 \hat{J}^{i, n}_t, \end{aligned}$$

where the $\hat{J}^{i, n}$ s are defined similarly to the $J^i$s in (6.4) but with $(\hat{\mathcal {X}}^n,\hat{X}^n,\hat{B}^n)$ in place of $(X,X^n,B)$. We want to let $n \rightarrow \infty$ in the above equality and recover (3.5) (equivalently (1.9)). For $\hat{J}^{0, n}_t$, we have

$$\begin{aligned} \sqrt{n}\hat{J}^{0, n}_t = \hat{V}^n_t \int _0^1 \nabla u\big (t, \hat{X}^n_t+ \vartheta (\hat{\mathcal {X}}^n_t-\hat{X}^n_t) \big ) \, d \vartheta , \end{aligned}$$

and by using the continuity of ∇u and the convergence in (6.17), we get by virtue of Lebesgue’s theorem on dominated convergence that $\sqrt{n}\hat{J}^{0, n}_t \rightarrow \nabla u (t, \hat{X}_t) \hat{V}_t$ in probability. Similarly, we obtain that

$$\begin{aligned} \sqrt{n}\hat{J}^{1, n}_t \xrightarrow {\hat{\textbf{P}}} \theta \int _0^t\nabla u(r,\hat{X}_r)\hat{V}_rdr. \end{aligned}$$

As in (6.12) and (6.13), we have that $\sqrt{n}\hat{J}^{2, n}_t \rightarrow 0$ and $\sqrt{n}\hat{J}^{3, n}_t \rightarrow 0$, in $L_p(\hat{\Omega })$, hence, also in probability. We move to $\hat{J}^{4, n}$. We have that

$$\begin{aligned} \sqrt{n}\hat{J}^{4, n}_t = \int _0^t \int _0^1 \nabla u (r, \hat{X}^n_r) \hat{V}^n_r \nabla \sigma \big (\hat{X}^n_r+\vartheta (\hat{\mathcal {X}}^n_r- \hat{X}^n_r) \big ) \, d \vartheta \, dr. \end{aligned}$$

By (6.17), combined with the continuity of $\nabla \sigma$ and ∇u (see (3.3)), by virtue of Lebegue’s dominated convergence theorem, we have that

$$\begin{aligned} \sqrt{n}\hat{J}^4_t \xrightarrow {\hat{\textbf{P}}} \int _0^t \nabla u (r, \hat{X}_r) \hat{V}_r \nabla \sigma \big (\hat{X}_r) \big ) \, dr. \end{aligned}$$

Next, we see that

$$\begin{aligned} \sqrt{n} \hat{J}^{5, n}_t&= \int _0^t\int _0^1 [I+ \nabla u(r, \hat{X}^n_r)] \nabla \sigma (\hat{X}^n_r-\vartheta (\hat{X}^n_r-\hat{X}^n_{\kappa _n(r)}))d\\  &\qquad \vartheta b(\hat{X}^n_{\kappa _n(r)}) \sqrt{n} (r-\kappa _n(r)) \, d\hat{B}^n_r \\  &\quad +\frac{1}{\sqrt{2}}\int _0^t\int _0^1 \nabla u(r,\hat{X}^n_r) \nabla \sigma (\hat{X}^n_r-\vartheta (\hat{X}^n_r-\hat{X}^n_{\kappa _n(r)}))d \vartheta \sigma (\hat{X}^n_{\kappa _n(r)}) d\hat{W}^n_r. \end{aligned}$$

Since $\nabla u,\nabla \sigma$ and *b* are bounded, the first term vanishes in the limit. For the second term, we can use the uniform continuity of $\nabla u,\sigma$, and $\nabla \sigma$ together with (6.17) to obtain, by virtue of Lemma A.1,

$$\begin{aligned} \sqrt{n} \hat{J}^{5, n}_t \xrightarrow {\hat{\textbf{P}}} \frac{1}{\sqrt{2}} \int _0^t \nabla u(r,\hat{X}_r) \nabla \sigma (\hat{X}_r) \sigma (\hat{X}_r) d\hat{W}_r. \end{aligned}$$

For the last term, we claim that

6.19 $$\begin{aligned} \sqrt{n} \hat{J}^{6, n}_t \xrightarrow {\hat{\textbf{P}}} \int _0^t \nabla ^2 u(r,\hat{X}_r) \hat{V}_r \sigma (\hat{X}_r) d\hat{B}_r. \end{aligned}$$

To see this, let p>(d+2)/2 and let $u_m$ be a smooth approximation of *u* such that $\Vert u_m -u\Vert _{L_{4p}([0, 1]; W^2_p)} \rightarrow 0$, as $m \rightarrow \infty$. For ε>0, we have

$$\begin{aligned} \,&\hat{\textbf{P}} \left( \Big | \sqrt{n} \hat{J}^{6, n}_t - \int _0^t \nabla ^2 u(r,\hat{X}_r) \hat{V}_r \sigma (\hat{X}_r) d\hat{B}_r \Big |> 3 \varepsilon \right) \\ \le \,&\hat{\textbf{P}} \left( \Big | \int _0^t \sqrt{n} \big [ (\nabla u - \nabla u_m)(r, \hat{\mathcal {X}}^n_r)- (\nabla u - \nabla u_m)(r, \hat{X}^n_r) \big ] \sigma (\hat{\mathcal {X}}^n_r) \, d\hat{B}^n_r \Big |> \varepsilon \right) \\&\, +\hat{\textbf{P}} \left( \Big | \int _0^t \sqrt{n} \big [ \nabla u_m(r, \hat{\mathcal {X}}^n_r)- \nabla u_m(r, \hat{X}^n_r) \big ] \sigma (\hat{\mathcal {X}}^n_r) \, d\hat{B}^n_r \right. \\&\left. - \int _0^t \nabla ^2 u_m (r,\hat{X}_r) \hat{V}_r \sigma (\hat{X}_r) d\hat{B}_r\Big |> \varepsilon \right) \\&\, +\hat{\textbf{P}} \left( \Big | \int _0^t \big [ \nabla ^2 u_m(r, \hat{X}_r)- \nabla ^2 u(r, \hat{X}_r) \big ] \hat{V}_r \sigma (\hat{X}_r) \, d\hat{B}_r\Big | > \varepsilon \right) \\ \, =&\, A_1^{n, m}+A_2^{n,m}+A_3^m . \end{aligned}$$

For $A_1$, we use Markov’s inequality, Itô’s isometry, the boundedness of σ, and (6.6) to obtain

$$\begin{aligned} A^{n, m}_1 \!\le \! \varepsilon ^{-2} \Vert \sigma \Vert _{C^0}\textbf{E}\! \int _0^t |V^n_r|^2 \! \Big ( \mathcal {M}| \nabla ^2 (u_m- u) | (r,\hat{\mathcal {X}}^n_r)\!-\! \mathcal {M}| \nabla ^2 (u_m\!-\! u) | (r,\hat{X}^n_r) \Big )^2 \, dr. \end{aligned}$$

Next, we use the the Cauchy-Schwartz inequality, the uniform bound on $\hat{V}^n$ which follows from Proposition 6.1 and (6.15) and Krylov’s estimate as well as its discrete version from Lemma 6.3, and (6.7), to see that

$$\begin{aligned} \sup _n A^{n, m}_1&\le \! N \varepsilon ^{-2} \Big (\textbf{E}\int _0^t\big ( \mathcal {M}| \nabla ^2 (u_m- u) | (r,\hat{\mathcal {X}}^n_r)\!-\! \mathcal {M}| \nabla ^2 (u_m- u) | (r,\hat{X}^n_r) \big )^4 \, dr \Big )^{1/2} \\&\le N \varepsilon ^{-2} \Vert \mathcal {M}| \nabla ^2 (u_m- u) |^ 4 \Vert _{L_p([0, 1] \times {\mathbb {R}}^d)}^{1/2} \\&\le N \varepsilon ^{-2} \Vert \nabla ^2 (u_m- u) \Vert _{L_{4p}([0, 1] \times {\mathbb {R}}^d)}^{2p} \rightarrow 0, \end{aligned}$$

as $m \rightarrow \infty$. In a similar manner, we get that $A^m_3 \rightarrow 0$ as $m \rightarrow \infty$. For $A^{n,m }_2$, we claim that for fixed $m \in \mathbb {N}$, $A^{n,m}_2 \rightarrow 0$, as $n \rightarrow \infty$. Indeed, for fixed *m*, since $u_m$ and σ are sufficiently regular, it is easy to see by (6.17) and the fundamental theorem of calculus that

$$\begin{aligned} \sup _{ r \in [0, 1]}| \sqrt{n} \big [ \nabla u_m(r, \hat{\mathcal {X}}^n_r)- \nabla u_m(r, \hat{X}^n_r) \big ] \sigma (\hat{\mathcal {X}}^n_r) \, - \nabla ^2 u_m (r,\hat{X}_r) \hat{V}_r \sigma (\hat{X}_r)| \xrightarrow {\hat{\textbf{P}}} 0. \end{aligned}$$

By the above combined with (6.17) and Lemma A.1, the claim follows. Consequently, we have shown (6.19).

Finally, we can now go back to (6.18) and let $n \rightarrow \infty$ in order to see that the processes $(\hat{X}, \hat{V}, \hat{B}, \hat{W})$ indeed satisfy (3.5), that is, (iv) from Definition 3.4 is satisfied. This finishes the proof. □

## Appendix A

The following is a straightforward consequence of [7, Theorem 2.2].

### Lemma A.1

For $n \in \mathbb {N}\cup \{0\}$, let $B^n$ be continuous $\mathbb {F}^n$-martingales and let $G^n$ be cádlág $\mathbb {F}^n$-adapted processes. Assume that $G^n \rightarrow G^0$ and $B^n \rightarrow B^0$, uniformly on [0, 1], in probability. Then, in probability, we have

$$\begin{aligned} \sup _{t \in [0, 1]}\big |\int _0^t G^n_s \, dB^n_s - \int _0^t G^0_s \, dB^0_s\big | \rightarrow 0. \end{aligned}$$

### Lemma A.2

Let *Y* be a process on [0, 1] and $\beta \in (0,1)$. For any $\varepsilon \in (0, \beta )$, there exists $p_0 \ge 0$ such that for all $p \ge p_0$ we have

$$\begin{aligned} [ Y ]_{L_p(\Omega ; C^{\beta -\varepsilon }([0, 1]))} \le N [ Y]_{C^{\beta }([0, 1];L_p(\Omega ))}, \\ \Vert Y\Vert _{L_p(\Omega ; C^{\beta -\varepsilon }([0, 1]))} \le N \Vert Y\Vert _{C^{\beta }([0, 1];L_p(\Omega ))}, \end{aligned}$$

where *N* depends only on p,β and ε.

The proof of the above lemma is a simple application of Kolmogorov’s continuity criterion or of the Sobolev Slobodeckij embedding theorem.

### Lemma A.3

Under Assumptions 2.1 and 3.1, estimate (3.4) holds.

### Proof

Throughout the proof, as usual, the constants *N* might change from line to line but only depends on $\lambda , \Vert b\Vert _{C^0({\mathbb {R}}^d)}, \Vert b\Vert _{L_m({\mathbb {R}}^d)}, d, \theta , \Vert \sigma \Vert _{C^2({\mathbb {R}}^d)}$, and α. Let $u^{(\ell )}$ be the solution of (1.7). By [9, Theorem 2.1] we have that

A.1 $$\begin{aligned} \Vert u\Vert _{L_{p_0}((0, 1); W^2_m({\mathbb {R}}^d))} \le N \Vert b^{(\ell )}\Vert _{L_{p_0}((0, 1); L_m({\mathbb {R}}^d)) } \le N \Vert b^{(\ell )}\Vert _{W^\alpha _m({\mathbb {R}}^d) } < \infty . \end{aligned}$$

Next, let us consider the auxiliary equation

A.2 $$\begin{aligned} \partial _t v+\frac{1}{2}a^{(i,j)}\partial ^2_{i,j} v=f, \qquad u(1, \cdot )= 0, \end{aligned}$$

for $f \in C^\infty _c((0, 1) \times {\mathbb {R}}^d )$. It is known that it admits a unique classical solution which in addition is three times continuously differentiable in space. By [9, Theorem 2.1] again, we have

$$\begin{aligned} \Vert v\Vert _{L_{p_0}((0, T);W^2_m({\mathbb {R}}^d))} \le N \Vert f\Vert _{L_{p_0}((0, T);L_m({\mathbb {R}}^d))}. \end{aligned}$$

Moreover, by differentiating the equation in the spatial variable, in a similar manner we obtain that

$$\begin{aligned} \Vert v\Vert _{L_{p_0}((0, T);W^3_m({\mathbb {R}}^d))} \le N \Vert f\Vert _{L_{p_0}((0, T);W^1_m({\mathbb {R}}^d))}. \end{aligned}$$

The above two estimates, by means of interpolation, imply that

A.3 $$\begin{aligned} \Vert v\Vert _{L_{p_0}((0, T);W^{2+\alpha }_m({\mathbb {R}}^d))} \le N \Vert f\Vert _{L_{p_0}((0, T);W^\alpha _m({\mathbb {R}}^d))}. \end{aligned}$$

Next, from (A.1), it follows that $u^{(\ell )}, b^{(\ell )}, b\cdot \nabla u^{(\ell )} \in L_{p_0}((0, T);W^{2+\alpha }_m({\mathbb {R}}^d))$. Hence, for $n \in \mathbb {N}$, there exists $g^n \in C^\infty _c((0, 1) \times {\mathbb {R}}^d)$ such that $g^n \rightarrow g:= \theta u^{(\ell )}-b^{(\ell )}-b\cdot \nabla u^{(\ell )}$ in $L_{p_0}((0, T);W^\alpha _m({\mathbb {R}}^d))$, an $n \rightarrow \infty$, and $\Vert g_n\Vert _{L_{p_0}((0, T);W^\alpha _m({\mathbb {R}}^d))} \le \Vert g\Vert _{L_{p_0}((0, T);W^\alpha _m({\mathbb {R}}^d))}$, for all $n \in \mathbb {N}$. Moreover, let $u_n$ be the solution of (A.2) with *f* replace by $g^n$. By (A.3), we have

$$\begin{aligned} \Vert u_n\Vert _{L_{p_0}((0, T);W^{2+\alpha }_m({\mathbb {R}}^d))}&\le N \Vert g_n\Vert _{L_{p_0}((0, T);W^\alpha _m({\mathbb {R}}^d))} \\&\le N \Vert \theta u^{(\ell )}-b^{(\ell )}-b\cdot \nabla u^{(\ell )} \Vert _{L_{p_0}((0, T);W^\alpha _m({\mathbb {R}}^d))} \\&\le N \Vert b^{(\ell )}\Vert _{W^\alpha _m({\mathbb {R}}^d) }, \end{aligned}$$

where for the last inequality we have used (A.1), Assumption 3.1, and the boundedness of $\nabla u^{(\ell )}$ from (3.2). Moreover, it is known by standard solvability theory that $(u_n,\nabla u_n, \nabla ^2 u_n) \rightarrow (u, \nabla u, \nabla ^2 u)$ almost everywhere in $(0, 1) \times {\mathbb {R}}^d$, as $n \rightarrow \infty$. Hence, the desired estimate follows from Fatou’s lemma.
